# A living organoid biobank of patients with Crohn’s disease reveals molecular subtypes for personalized therapeutics

**DOI:** 10.1016/j.xcrm.2024.101748

**Published:** 2024-09-26

**Authors:** Courtney Tindle, Ayden G. Fonseca, Sahar Taheri, Gajanan D. Katkar, Jasper Lee, Priti Maity, Ibrahim M. Sayed, Stella-Rita Ibeawuchi, Eleadah Vidales, Rama F. Pranadinata, Mackenzie Fuller, Dominik L. Stec, Mahitha Shree Anandachar, Kevin Perry, Helen N. Le, Jason Ear, Brigid S. Boland, William J. Sandborn, Debashis Sahoo, Soumita Das, Pradipta Ghosh

**Affiliations:** 1 Department of Cellular and Molecular Medicine, University of California, San Diego, La Jolla, CA 92093, USA; 2 HUMANOID™ Center of Research Excellence (CoRE), University of California, San Diego, La Jolla, CA 92093, USA; 3 Department of Computer Science and Engineering, Jacobs School of Engineering, University of California, San Diego, La Jolla, CA 92093, USA; 4 Department of Pathology, University of California, San Diego, La Jolla, CA 92093, USA; 5 Department of Medicine, University of California, San Diego, La Jolla, CA 92093, USA; 6 Department of Pediatrics, University of California, San Diego, La Jolla, CA 92093, USA

**Keywords:** patient-derived organoids, inflammatory bowel disease, barrier integrity, host-microbe interaction, therapeutics

## Abstract

Crohn’s disease (CD) is a complex and heterogeneous condition with no perfect preclinical model or cure. To address this, we explore adult stem cell-derived organoids that retain their tissue identity and disease-driving traits. We prospectively create a biobank of CD patient-derived organoid cultures (PDOs) from colonic biopsies of 53 subjects across all clinical subtypes and healthy subjects. Gene expression analyses enabled benchmarking of PDOs as tools for modeling the colonic epithelium in active disease and identified two major molecular subtypes: immune-deficient infectious CD (IDICD) and stress and senescence-induced fibrostenotic CD (S2FCD). Each subtype shows internal consistency in the transcriptome, genome, and phenome. The spectrum of morphometric, phenotypic, and functional changes within the “living biobank” reveals distinct differences between the molecular subtypes. Drug screens reverse subtype-specific phenotypes, suggesting phenotyped-genotyped CD PDOs can bridge basic biology and patient trials by enabling preclinical phase “0” human trials for personalized therapeutics.

## Highlights

• Prospectively biobanked CD organoids recapitulate the disease epithelium in patients
• The phenome-transcriptome-genome of CD organoids converges on two molecular subtypes
• One subtype shows impaired microbial clearance, and another increased cellular senescence
• Phenotyped-genotyped PDOs are then used for integrative and personalized therapeutics

## Graphical abstract

**Figure:**
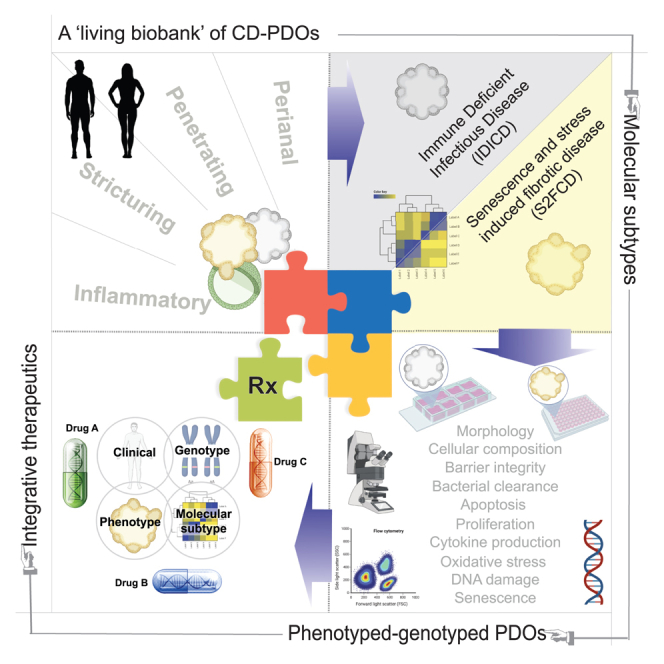

## Teaser

Tindle et al. prospectively created a biobank of phenotyped-genotyped Crohn’s disease patient-derived organoids (CD PDOs), identifying two distinct molecular subtypes. This work underscores the potential of CD PDOs as platforms for leveraging molecular classification to advance personalized therapeutics, effectively bridging the gap between basic research and clinical trials.

## Introduction

Crohn’s disease (CD) is a chronic incurable disease.^1^ It is characterized by relentless progression with complications, e.g., intestinal fibrosis, penetrating fistulas, and bowel destruction, that are fueled by inflammation.^2^ The unremitting inflammation in CD is believed to be multifactorial in origin.^2^ Dysregulated interactions between the luminal microbiome, host genome, environmental triggers, and the gut immune system have been implicated. Although genetics explains some variation in ileal vs. ileo/colonic-predominant location of CD,^3^ it does not rationalize disease extent, behavior, therapeutic response, predisposition to environmental triggers (infection, smoking), or risk of complications (e.g., extraintestinal manifestations and risk of colorectal cancers [CRCs]).^3^ CD also lacks good preclinical animal models that faithfully recapitulate the diverse components of the human diseased tissue. Thus, CD continues to pose a challenge, and insights that can translate into personalization and precision in patient care are slow to emerge.

**Figure 1:**
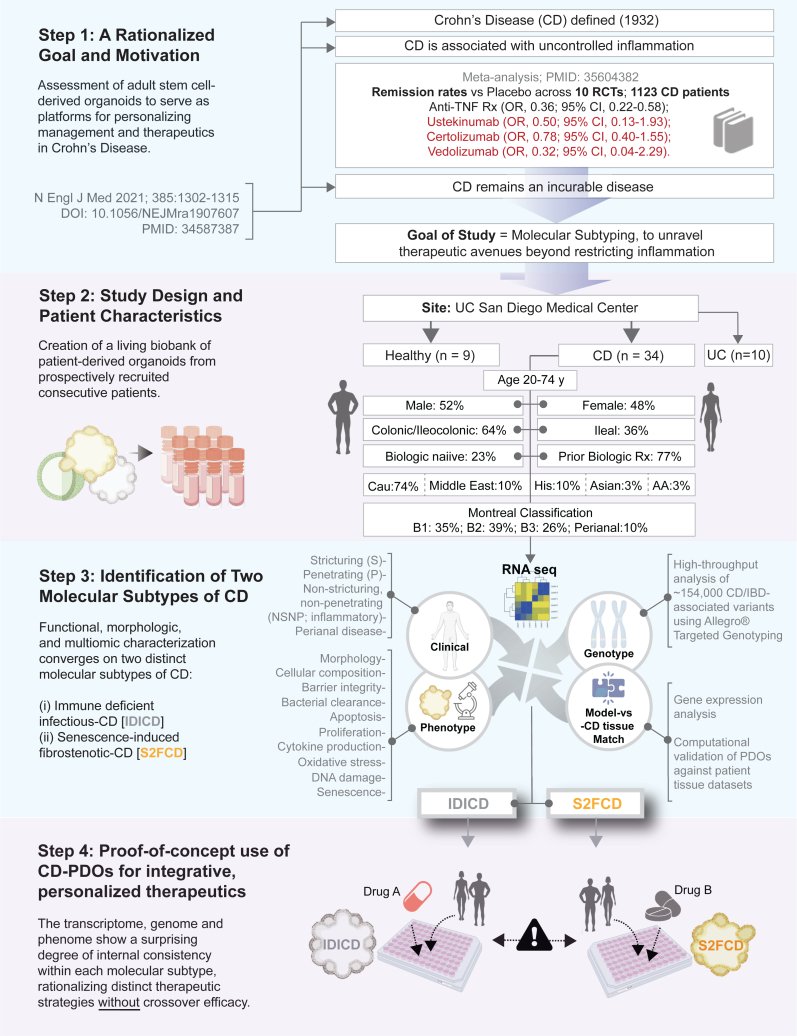
Study outline: Creation of a living biobank of adult stem cell-derived PDOs for enhancing personalized therapeutics in CD Key aspects of a rationalized goal and study motivation are summarized (Step 1). Patients were prospectively enrolled in this study as source of colonic tissue biopsies for the isolation and creation of the CD PDO biobank. Clinical, pathological, and treatment history and the Montreal classification of disease were collected (see Table S1). PDOs were generated from the adult stem cells at the crypt base, expanded, and biobanked (in Step 2) for use in various assays (in Step 3). Various multiomic, morphologic, and functional studies that were performed, some in low- and others in high-throughput modes (HTP; in 96-well plates) for systematic molecular and phenotypic characterization (cataloged in Table S2). The study ends with proof-of-concept therapeutic studies (Step 4) in which drugs are rationalized and paired to each subtype with the intention to specifically reverse driver phenotyped within a subtype without crossover benefits to the other subtype. RCT, randomized controlled trials; OR, odds ratio; CI, confidence interval.

Recently, numerous studies have begun to unravel the role of different gut epithelial cell types using human patient-derived organoids (PDOs).^4^^,^^5^ These studies show that PDOs may faithfully recapitulate phenotypes in both CD ileum^6^ and ulcerative colitis (UC, another variant of inflammatory bowel diseases, IBD) colon,^7^ transcriptome and secretome,^8^ the presence of stem cell^9^^,^^10^ and telomere dysfunction,^11^ increased apoptosis,^12^ and impaired wound healing^13^ and barrier function.^14^^,^^15^ Most of these studies suffer from limited number of unique subjects or limited focus, i.e., UC colon or CD ileum. None comprehensively studied the major clinical subtypes or disease behavior (Montreal classification^16^; B1-inflammatory, B2-stricturing, and B3-penetrating). Consequently, the molecular basis for such diverse clinical presentation has remained elusive and most of the current Food and Drug Administration (FDA)-approved therapeutic options^1^ lack personalization and, unsurprisingly, fail to achieve and/or maintain remission, especially in B3-penetrating/fistulizing disease^17^ (Figure 1; *Step 1*). Use of anti-inflammatory drugs may also be imprecise; they target proinflammatory cytokines while failing to tackle fundamental triggers that initiate and perpetuate inflammation.

Here we sought to address some of these challenges by prospectively creating a large living biobank of CD PDOs generated from colon biopsies (*n* = 53; 34 CD PDOs, 10 UC-PDOs, sampled for inflamed vs. uninflamed locations of 4 subjects and 9 healthy PDOs) representative of the diverse patient population receiving care at any specialized tertiary care center in the United States (Figure 1; *Step 2*). Our approaches were geared to establish a way of “benchmarking” CD PDOs, through objective and stringent criteria, as reproducible and close “replica” of the epithelial dysfunction in the diseased tissue (Figure 1; *Step 3*). Our findings highlight the potential of CD PDOs to enable molecular subtyping of the disease and the use of phenotyped-genotyped CD PDOs as platforms for preclinical phase “0” trials for personalized and integrative therapeutics (Figure 1; *Step 4*).

## Results and discussion

### Establishment of a living CD biobank: Study rationale and outline

Consecutive patients presenting to the UC San Diego IBD Center were enrolled in this study. The only criteria were a clinically confirmed diagnosis of CD as the indication for an endoscopy and the ability to obtain informed consent. Patient characteristics are detailed in Table S1 and summarized in Figure 1, *Step 2*. All the major CD behaviors, as per the Montreal classification,^16^ including the perianal modifier, were adequately represented (Figure 1; *Step 2*).

We focused on the colon because of the following reasons. (1) It is involved in ∼60% of patients with CD; while ∼ half of those patients have synchronous involvement of the small intestine, the other half show disease that is limited to the colon.^18^ (2) PDOs derived from the ileum had thus far failed to reveal disease subtypes. We chose to use adult stem cells to derive the organoids (as opposed to induced pluripotent stem [iPS] cells) because we wanted to capture the disease-driving epigenetics and not just genetics.

To generate the biobank of PDOs, we adapted the culture conditions for long-term expansion of human colonic epithelium from *LGR5*-positive stem cells located at the bottom of the colon crypts as done previously, with a few modifications.^19^^,^^20^^,^^21^^,^^22^^,^^23^^,^^24^^,^^25^^,^^26^ We used L-WRN conditioned media^20^^,^^21^^,^^22^ (instead of defined media) because it contains biologically active Wnt (*W*; a ligand that is necessary for maintaining active crypt stem cells^27^^,^^28^^,^^29^), R-Spondin1 (*R*; the ligand for LGR5^30^), and Noggin (*N*) and in consistent proportions and has previously been shown to yield reproducible results for PDO cultures across laboratories.^23^ All organoids could be readily expanded and frozen to create a living biobank with 100% success rate. Upon thawing, cell survival was typically >90%, allowing us to systematically analyze them using “omics,” morphological, and functional studies, some of which were performed in low- and several in high-throughput (HTP) modes. Integration of this information led to the identification of two distinct molecular subtypes of CD (Figure 1; *Step 3*). This revelation inspired the use of PDOs as preclinical models to pair therapeutics that are specific to the molecular subtype, with the goal to reverse key disease-driving cellular process(es) (Figure 1; *Step 4*).

### Objectivity in benchmarking PDOs as reproducible “replica” of the diseased epithelium

**Figure 2:**
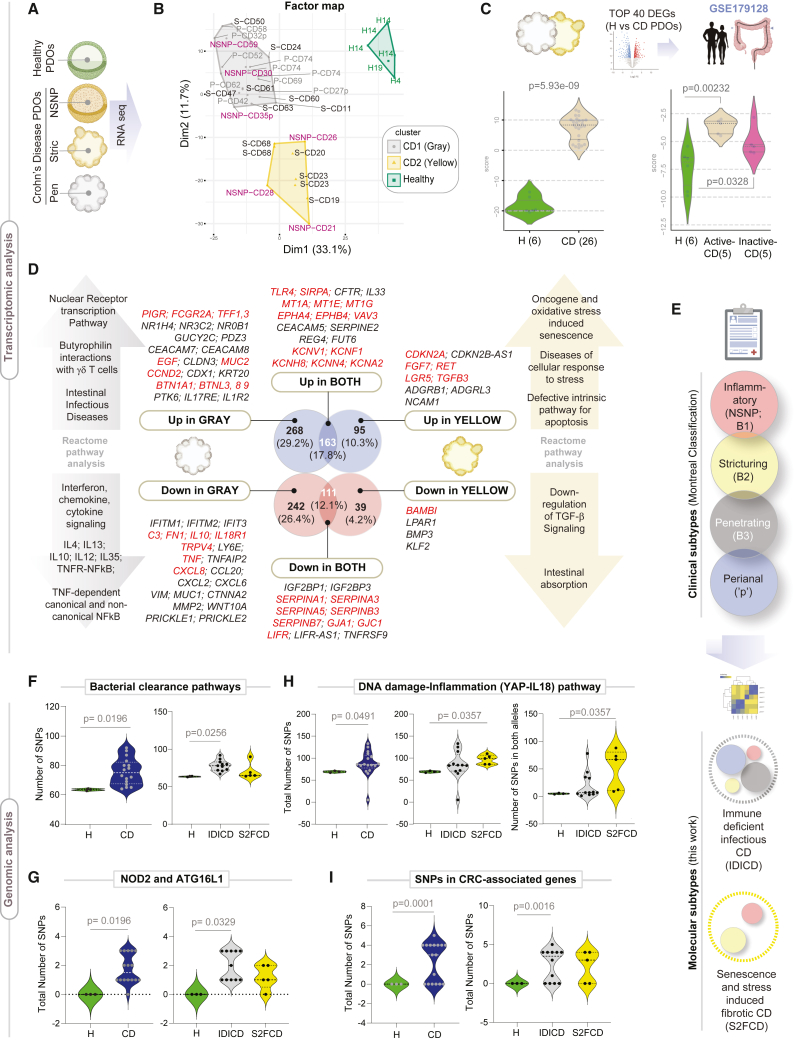
Transcriptome and genome analyses of CD PDOs reveal the existence of two distinct molecular subtypes of CD (A) Study design for the transcriptomic analyses on PDOs. (B) A factorial map generated by performing the hierarchical clustering on principal components (HCPC) analysis is plotted onto the first two dimensions. The CD PDOs cluster into two distinct groups: gray and yellow, which are differentiated from healthy controls (green). Individual samples are labeled (see Table S3 for patient information). H, healthy; NSNP, non-stricturing, non-penetrating; S, stricturing; P, penetrating. Samples (annotated with “p”) were from subjects with perianal disease. See also Figure S1 for comparison with UC-PDOs. (C) Top: the strategy used to objectively benchmark CD PDOs. Bottom: violin plots show the composite score of the PDO-derived top upregulated DEGs (left) and in the laser capture microdissected colonic epithelium (right). Values in parentheses indicate unique patients. See Table S4 for the list of DEGs. (D) Genes that are differentially expressed (Up or Down vs. healthy) uniquely in the gray (CD1; left) vs. yellow (CD2; right) cluster of CD PDOs or those that are shared between both subtypes (CD1+2) are listed, alongside the enriched pathways they represent. See Table S4 for a complete list of DEGs and reactome pathways. See also Figures S2 and S3 for additional principal component analysis (PCoA) and reactome pathway enrichment analyses and Figures S4 and S5 for transcriptome-derived insights into CD-associated changes in the crypt-axis differentiation score, stem cell dysfunction, cellular composition, and other properties. (E) Schematic summarizes how various clinical subtypes of CD (Montreal classification) fit into two broad molecular subtypes, immune-deficient infectious CD and senescence and stress-induced fibrotic CD. (F–I) Violin plots show the number of mutations in genes within the indicated pathways (see Table S5 for gene list) in CD PDOs vs. healthy controls. Plots on the left compare all CD PDOs combined, whereas plots on the right separate the CD PDOs by molecular subtypes, IDICD and S2FCD. Plots in (G) specifically display the frequency of NOD2 SNPs rs2066843 and rs2076756 and ATG16L1 SNP rs2241880 in CD PDOs vs. healthy controls. Statistical significance was assessed by Mann-Whitney (F, G [left], H), one-way ANOVA (G, right), and Welch’s test (I). Only significant *p* values are displayed. See also Figure S6.

First, we benchmarked PDOs by RNA sequencing (RNA-seq) to assess if they retain the altered gene expression pattern in the epithelium of the IBD-afflicted colon (Figures 2A–2D; see Table S3). Principal-component analyses (PCAs) showed that, while the healthy and CD PDOs were distinct from each other, the CD PDOs segregated into two distinct clusters (CD1-gray and CD2-yellow; Figure 2B). All the penetrating (P, B3) CD PDOs and PDOs from the perianal disease were in the gray cluster (Table S3); however, the NSNP (B1) and stricturing (B2) CD PDOs were split between the gray and the yellow clusters (Figure 2B). UC-PDOs co-clustered with CD PDOs in the gray cluster (Figures S1A and S1B), regardless of whether they were derived from the involved or uninvolved segments of the colon, indicating that UC may have shared pathophysiology with one of the two CD subtypes, but both are distinct from the healthy PDOs. An analysis of differentially expressed genes (DEGs) revealed the common set of genes and cellular pathways and processes that are up- or downregulated in both the gray and yellow clusters (Table S4 for the list of DEGs and pathways).

To objectively assess how well PDOs reproduced the altered epithelial biology in the setting of active disease, we took advantage of a high-quality publicly available dataset of laser microdissected IBD-afflicted colonic epithelia (GSE179128)^31^; as one of a kind, this dataset included both active and inactive states of UC and CD. The top upregulated DEGs in CD PDOs were induced in the micro-dissected CD-afflicted (Figure 2C) and UC-afflicted (Figure S1C) colonic epithelia, exclusively in the active disease state. Similarly, the top upregulated DEGs in UC-PDOs were induced in the micro-dissected IBD-afflicted colonic epithelia exclusively in the active disease state (Figure S1D). These unbiased assessments of the “match” between gene expression patterns in PDO vs. tissue indicated that the CD/UC-PDOs replicate the disease activity in the colonic epithelium of patients. When IBD organoids generated by other groups were analyzed using the same unbiased yardstick of gene expression patterns in the colonic epithelium, some reproduced the disease state, but others did not: reproducibility was seen in studies where adult stem cells from the colon crypt were used to derive the PDOs and L-WRN conditioned media was used for their expansion and biobanking^32^^,^^33^ (Figure S1E) but PDOs grown in defined media was lacking^11^^,^^24^ or where PDOs were derived from iPS cells^34^ (Figures S1F and S1G).

### Identification of two distinct molecular subtypes of CD

Next, we compared the gene expression patterns in the gray vs. yellow clusters (Figure 2D; see Table S4 for DEGs and reactome pathways; Figures S2 and S3 for PCA, heatmap, and reactome analyses). Uniquely upregulated in the gray cluster were genes that are involved in intestinal infectious diseases and the butyrophilins; the latter are molecules that are used by epithelial cells to shape organ-specific γδ T cells^35^ (Figure 2D, *top left*). One notably upregulated gene is the polymeric immunoglobulin receptor (*PIGR*), mutations in which have been implicated in an increased risk for IBD.^36^^,^^37^^,^^38^ Uniquely downregulated in the gray cluster were genes that are involved in all the major interferon (IFN), chemokine, and cytokine signaling pathways (Figure 2D, *bottom left*). As for the yellow cluster, the uniquely upregulated genes were, among others, *CDKN2A*, *LGR5*, and *RET*, all known to be involved in oncogene and oxidative stress-induced senescence, cellular response to stress, and defects in apoptosis (Figure 2D, *top right*). Uniquely downregulated in the yellow cluster were genes such as *BAMBI*, the potent endogenous inhibitor of fibrogenic transforming growth factor β signals^39^ (Figure 2D, *bottom right*). The upregulated DEGs shared between the two subtypes were notable for the pattern recognition receptor, *TLR4*, and its negative regulator and the self-recognition molecule, Signal Regulatory Protein Alpha (*SIRPA*); metallothioneins, *MT1A*, *MT1E*, and *MT1G*; the Ephrins *EPHA4* and *EPHB4*; voltage-gated potassium channels; and the RhoGTPase and oncogene, *VAV3* (Figure 2D). The downregulated DEGs shared between both subtypes were notable for numerous serine protease inhibitor genes (*SERPINEA1*, *-A3*, *-A5*; *-B3*, *-B7*) and gap junction genes *GJA1* and *GJC1*. We also observed the downregulation of *TNFSF9*, which is known to enhance epithelial tight junction (TJ) resistance.^40^

These results—reproduced in two sets of recruitments during the study—indicate that despite clinical heterogeneity (Montreal classification into 4 behaviors; Figure 2E, *top*), PDOs generally fit into one of two molecular subtypes (Figure 2E, *bottom*). Because the gray cluster uniquely represented an infectious disease-like state in the setting of reduced cytokine/inflammatory responses (i.e., paradoxical immune deficiency), we named this subtype as an immune-deficient infectious CD (IDICD). Because the yellow cluster uniquely represented stress and senescence in the setting of reduced anti-fibrogenic signaling, we named this subtype senescence and stress-induced fibrotic CD (S2FCD).

### Transcriptome reveals shared and distinct epithelium-intrinsic defects in CD molecular subtypes

Next we sought insights into the two molecular subtypes with gene signatures previously established through single-cell sequencing (scSeq) of the IBD mucosa. A 15-gene crypt-axis score^41^ (Figure S4A) revealed that the CD PDOs are skewed toward differentiation, in both 3D (Figure S4B, *left*) and 2D (Figure S4C) growth conditions; differentiation was more pronounced in IDICDs (Figure S4B, *right*). Consistently, *CEACAM7*+ terminally differentiated colonocytes^10^ were increased in CD PDOs, and the IDICD PDOs uniquely accounted for this (Figure S4D, *left*; Figure S4E). A comprehensive suite of previously established *LGR5*+ intestinal stem cells (ISC) signatures^42^ revealed that MHCII^+^ *Lgr5*^+^ ISCs are downregulated in both subtypes of CD PDOs, but more significantly in S2FCD (Figures S4F and S4G). Because MHCII^+^ *Lgr5*^+^ ISCs are non-conventional antigen-presenting cells for CD4^+^ T helper cells, constituting stem cell-immune cell synapses that balance self-renewal and differentiation in the setting of infection and inflammation,^42^ their reduction suggests a defect in this homeostatic pathway.

Analysis of the abundance of goblet (*MUC2*) and Paneth cell (*LYZ*, *REG3A*) transcripts suggested that the proportions of these cells may be altered (Figures S5A and S5B). Both CD subtypes were deficient in *WFDC2* (Figure S5C)—an antiprotease molecule that is expressed by goblet cells, inhibits bacterial growth, preserves TJ integrity, prevents bacterial invasion and suppresses mucosal inflammation, and is downregulated in the IBD colon.^41^ Both CD subtypes showed reduced TJ genes (Figure S5D) and DNA damage genes (Figure S5E), but S2FCD PDOs selectively showed reduction in the mitotic checkpoint complex genes (Figure S5F). The DEGs between healthy and CD PDOs could successfully classify treatment responders from non-responders (Figure S5G) even if the samples were collected prospectively (Figure S5H) and regardless of treatment modality (Figures S5H and S5I). These results suggest that the CD PDO-derived intrinsic epithelial processes may have predictive value.

To investigate if the transcriptome of the CD PDOs reflects the previously known genes/risk alleles implicated in CD, we first defined a list of genes nearest to every genetic variant significantly associated with CD based on genome-wide association studies (GWASs) (see STAR Methods; Figure S5J). Levels of expression of these set of genes were generally suppressed in CD PDOs (Figure S5J, *left*), and such suppression was significant in the IDICD PDOs (Figure S5J, *right*). A comprehensive analysis revealed that many of these genes were uniquely dysregulated in one or the other CD subtype (Figure S5K), suggesting IBD risk alleles that confer transcriptome changes may be uniquely reflected in CD subtypes.

### The transcriptome and genome converge within each molecular subtype of CD

Because genetic contributions to clinical CD subtypes remain elusive^3^ (Figure S6A), we asked how it may contribute to the molecular subtypes. We analyzed genomic DNA from healthy and CD PDOs for ∼154,000 IBD-associated SNPs by targeted sequencing (see STAR Methods; Figure S6B). SNPs in genes within the bacterial clearance pathway (see Table S5) were over-represented in the CD PDOs, specifically in the IDICD subtype (Figure 2F). More specifically, CD risk alleles in *NOD2* and *ATG16L1* were over-represented in the IDICD CD PDOs (Figure 2G). By contrast, SNPs in genes within a senescence- and DNA-damage-induced *YAP-IL18* proinflammatory pathway^43^ were over-represented in the S2FCD PDOs (Figure 2H). CRC-associated mutations were over-represented in the CD PDOs, primarily in the IDICDs (Figure 2I); these results agree with epidemiologic studies showing higher CRC risk in penetrating (B3)^44^^,^^45^ and perianal fistulizing CD.^46^ A higher frequency of SNP was also found in monogenic risk alleles—*RET* (Figure S6C) and *POU5F1* (Figure S6D); both genes have been associated with early-onset CD.^47^^,^^48^ *RET* supports cell-cell adhesion to resist tumor necrosis factor alpha (TNF-α) challenge,^49^ and mutations in this gene have been implicated in the development of enterocolitis independent of NOD2.^50^ Although CD PDOs harbored more mutations in genes associated with apoptosis (Figure S6E), DNA damage (Figure S6F), proliferation (Figure S6G), and epithelial TJs (Figure S6H), subtype specificity was not observed.

Because the SNPs that impair bacterial clearance are enriched in the IDICD subtype, and the SNPs within the senescence-associated DNA damage and inflammation are enriched in the S2FCD subtype (see Table 1), we conclude that the transcriptome and genome converge on related themes within each molecular subtype.

**Table 1:** A summary of major statistically significant findings reported in this work

Molecular subtype	IDICD (Immune-deficient infectious CD)				S2FCD (senescence- and stress-induced fibrotic CD)		Approach	Comments
Montreal classification	Penetrating (all)	NSNP (some)	Perianal (all)	Stricturing (few)	Stricturing (most)	NSNP (some)	RNA-seq analysis of CD PDOs vs. Healthy	N/A
Barrier integrity	Intact	Impaired	Intact	Intact in most, impaired in some	Intact in most, impaired in some	Impaired	TEER measurement ∗ FITC-dextran leakage ∗	∗ = HTP in 96-wells
Dysmorphic growth	No	Yes; defect in lumen formation	No	Yes; asymmetry, high cellularity	Yes; asymmetry, high cellularity	Yes	Light microscopy∗, IMARIS imaging∗	∗ = HTP in 96-wells
Apoptosis	High	Normal	NT	High (easily seen by H&E)	High	Normal	TUNEL assay#	# = LTP-semi-HTP in 8-wells
Apoptosis in response to TNF-α	High	High	NT	High	High	High		
Proliferation	High	Normal	NT	High	High	normal	BrdU incorporation ∗	∗ = HTP in 96-wells
Ox DNA/RNA damage	Normal	High	NT	High	High	High	ELISA-based assay ∗	∗ = HTP in 96-wells
Crypt cell composition, differentiation	High crypt-axis score, high *CEACAM7*+ brush border cells, high goblet cells; defective MHC-II+ ISC-III, high goblet cells				Low crypt-axis score, defective MHC-II+ ISC-III, high goblet cells; defect in mitotic checkpoint.		RNA-seq	N/A
Paneth cell dysfunction	Yes (excessive degranulation)				Yes (excessive degranulation)		Imaging (IF and EM)	LTP
Goblet cell AMP production	Impaired severely				Reduced slightly		RNA-seq	N/A
DNA damage (dsDNA break)	Normal				High		Flow cytometry (gH2AX)	LTP
Senescence	Normal				High		SPIDER β-Gal assay, Flow cytometry	# = LTP-semi-HTP in 8-wells
Bacterial clearance	Impaired				NT		Infection, lysis, plating and colony counting	LTP on EDMs
Induction of ROS	High				Very high		ELISA ∗	∗ = HTP in 96-wells
Microbe-challenged production of cytokines	Impaired				Normal		Multiplex ELISA assays (Mesoscale discovery) ∗	∗ = HTP in 96-wells
Genotype-phenotype relationship	SNPs in genes within the bacterial clearance pathway, NOD2/ATG16L1 disease-driving SNPs, and SNPs in CRC-associated genes.				SNPs in the genes within the DNA damage-YAP-IL18 inflammatory pathway.		Genotyping ∗ and SNP analysis	HTP

NSNP, non-stricturing non-penetrating; ROS, reactive oxygen species; NT, not tested; HTP, high throughput; LTP, low throughput.

### The molecular subtypes of CD PDOs display shared and distinct phenomes

We next asked if the convergent transcriptome and genome support a cohesive phenome in the CD PDOs. If so, such transcriptome-genome-phenome convergence could help rationalize therapeutic agents personalized to the molecular CD subtype and enable tracking of the phenome as an objective metric of therapeutic response. We tested this concept using the two molecular subtypes of CD PDOs.

#### S2FCD PDOs show dysmorphic growth features

**Figure 3:**
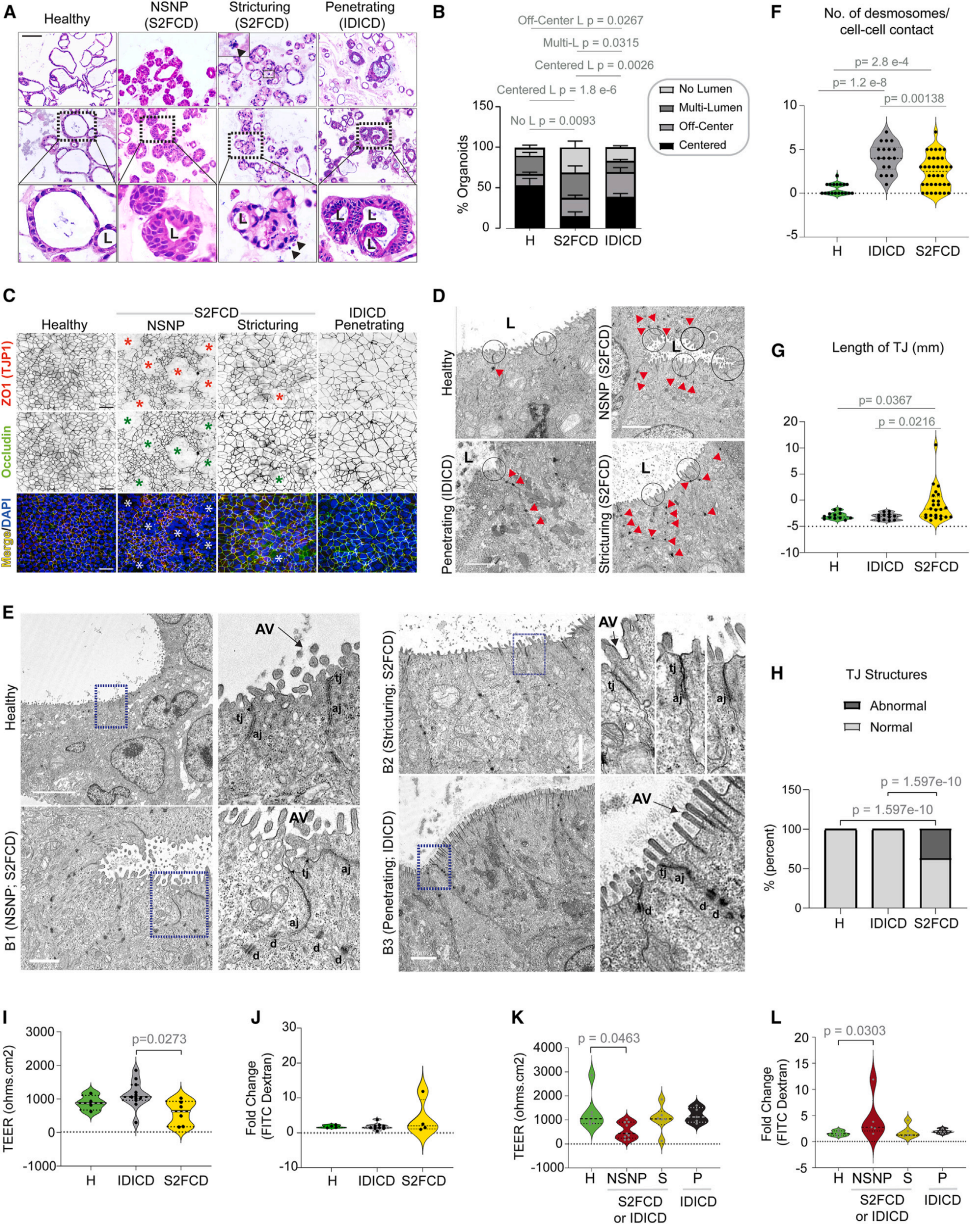
Assessment of morphology and barrier integrity of CD PDOs (A and B) Representative images (A) of hematoxylin and eosin-stained FFPE-CD PDOs are shown from each clinical subtype of CD alongside healthy controls. Scale bar, 100 μm. L, lumen. Arrowhead, nuclear fragmentation (likely apoptotic bodies). Stacked bar plots (B) show the quantification of the proportion of each type of organoid structure in various CD subtypes (See Figure S7B for all CD subtypes combined; B [right], separated into CD subtypes). Statistical significance was assessed by one-way ANOVA. Only significant *p* values are displayed (*n* = 3–8 in each group). See Figure S7 for morphologic assessment by quantitative morphometrics using Imaris and lumen position/presence by light microscopy. (C) Polarized monolayers of CD PDOs on transwells (enteroid-derived monolayers; EDMs) were fixed and stained for ZO1 (red), occludin (green), and DAPI (nuclei, blue) and analyzed by confocal microscopy. Representative fields are shown; individual red and green channels are displayed in grayscale. Asterisk, areas of impaired barrier. Scale bar, 50 μm. (D) Electron micrographs of healthy and CD PDOs display apical cell-cell junctions. Red arrowheads, desmosomes. Scale bar, 2 μm. (E) Electron micrographs of tight junctions in healthy and CD PDOs of various subtypes are shown. The boxed region on the left is magnified on the right. AV, apical villi; tj, tight junction; aj, adherens junction; d, desmosomes. Scale bars, 5 μm (top two panels) and 2 μm (bottom two panels). (F–H) Plots display the quantification of no. of desmosomes/cell-cell contact (F), the length of TJ (G), and the frequency of abnormal defects/TJ structure (H) observed by TEM. Statistical significance was assessed by Welch’s t test (F, G) and Fisher’s exact test (H) (*n* = 7–13 fields analyzed in each subtype of PDO). See Figures S8A–S8C for the same analysis displayed as clinical subtypes of CD. (I–L) Violin plots show the fold change in TEER across (I and K) and FITC-dextran leakage through (J and L) CD-EDMs compared to healthy EDMs. Data are displayed either as molecular (I and J) or clinical (K and L) subtypes. Statistical significance was assessed by Welch’s t test (I–K) and Mann-Whitney (L). Only significant *p* values are displayed (*n* = 5–9 subjects in each group, 2–5 repeats in each PDO). See Figures S8D–S8F for all CD subtypes combined. See Table S2 for subjects analyzed in each assay.

Unlike healthy colonoids, CD PDOs presented with a range of patient-specific morphologies in 3D growth conditions, as determined by light microscopy (Figures 3A and S7A). We observed that compared to their healthy counterparts, CD PDOs were less likely to grow as thin-walled organoids with a single central lumen and more likely to grow into compact structures without lumen (Figure S7B, *left*). S2FCD showed the most dysmorphic growth, whereas IDICD PDOs were the closest to healthy PDOs (Figure 3B). A clinical subgroup analysis showed that dysmorphic growth was the highest in both NSNP (B1) and stricturing (S, B2) CD PDOs, and least in the penetrating (P, B3) CD PDOs (Figure S7B, *right*). Findings by light microscopy were verified by quantitative fluorescence microscopy (Figure S7C); NSNP (B1) and stricturing (S, B2) CD PDOs consistently showed the most volume in 3D (vortex count, Figure S7D), higher cellularity (nuclear count; Figure S7E), and asymmetry (bounding box analyses; Figures S7F and S7G), whereas ellipticity and sphericity were comparable across all healthy and CD PDOs (Figures S7H and S7I). H&E staining also revealed the presence of frequent apoptotic nuclear events, which were unique to the stricturing (S, B2) CD PDOs (Figure 3A*, arrows*).

#### Inflammatory CD PDOs in the S2FCD molecular subtype display barrier dysfunction

To assess barrier integrity, we prepared enteroid-derived monolayers (EDMs) from healthy and CD PDOs using established methodologies^22^^,^^51^^,^^52^ that were previously successfully adapted to IBD-PDOs.^20^^,^^21^ We analyzed stably polarized EDMs (i.e., trans-epithelial electrical resistance [TEER] reaches plateau over 3 consecutive readings) for TJs by confocal microscopy (Figure 3C) and transmission electron microscopy (TEM) (Figures 3D–3H) and for paracellular permeability using two well-established functional assays, leakage of fluorescently labeled dextran^53^ and TEER^54^ (Figures 3I–3K). The TJ proteins, ZO1 and occludin, were readily visualized in EDMs from all CD subtypes; while 80% of NSNP (B1) and ∼25%–30% of stricturing (S) CD-EDMs showed breaks in the monolayer (asterisk, Figure 3C), none of the penetrating (IDICD) EDMs showed the same. TEM revealed an increased number of desmosomes in both IDICD and S2FCD subtypes (Figures 3D and 3E). The TJs were short, and the membrane leaflets of the neighboring cells were tightly opposed in healthy PDOs (Figure 3D); however, they were either elongated or they showed interrupted membrane opposition in the S2FCD PDOs (Figures 3C and 3D). As for the IDICD PDOs, the TJs and AJs appeared morphologically indistinguishable from healthy PDOs, with two notable exceptions: (1) desmosomes were increased and (2) apical microvilli were elongated and prominent in brush border cells (Figure 3D). Quantification of these features confirmed that these aberrant junctions were more frequently encountered in the S2FCD molecular subtype of PDOs (Figures 3E–3G) and the NSNP clinical subtype was the primary contributor to these observed aberrations (Figures S8A–S8C).

Although paracellular permeability was not increased in CD-EDMs compared with healthy EDMs (Figures S8D–S8F), a molecular subtype analysis confirmed that S2FCD PDOs are slightly leakier than IDICD PDOs, apparent only by TEER assessment (Figures 3I and 3J). Similarly, a clinical subtype analysis confirmed that NSNP (B1) EDMs are significantly leakier than healthy EDMs based on both lower TEER assessments (Figure 3K) and the recovery of higher amounts of fluorescein isothiocyanate (FITC)-dextran from the basolateral side (Figure 3L).

#### IDICD harbors Paneth cell defects, whereas S2FCD shows oxidative stress and DNA damage

**Figure 4:**
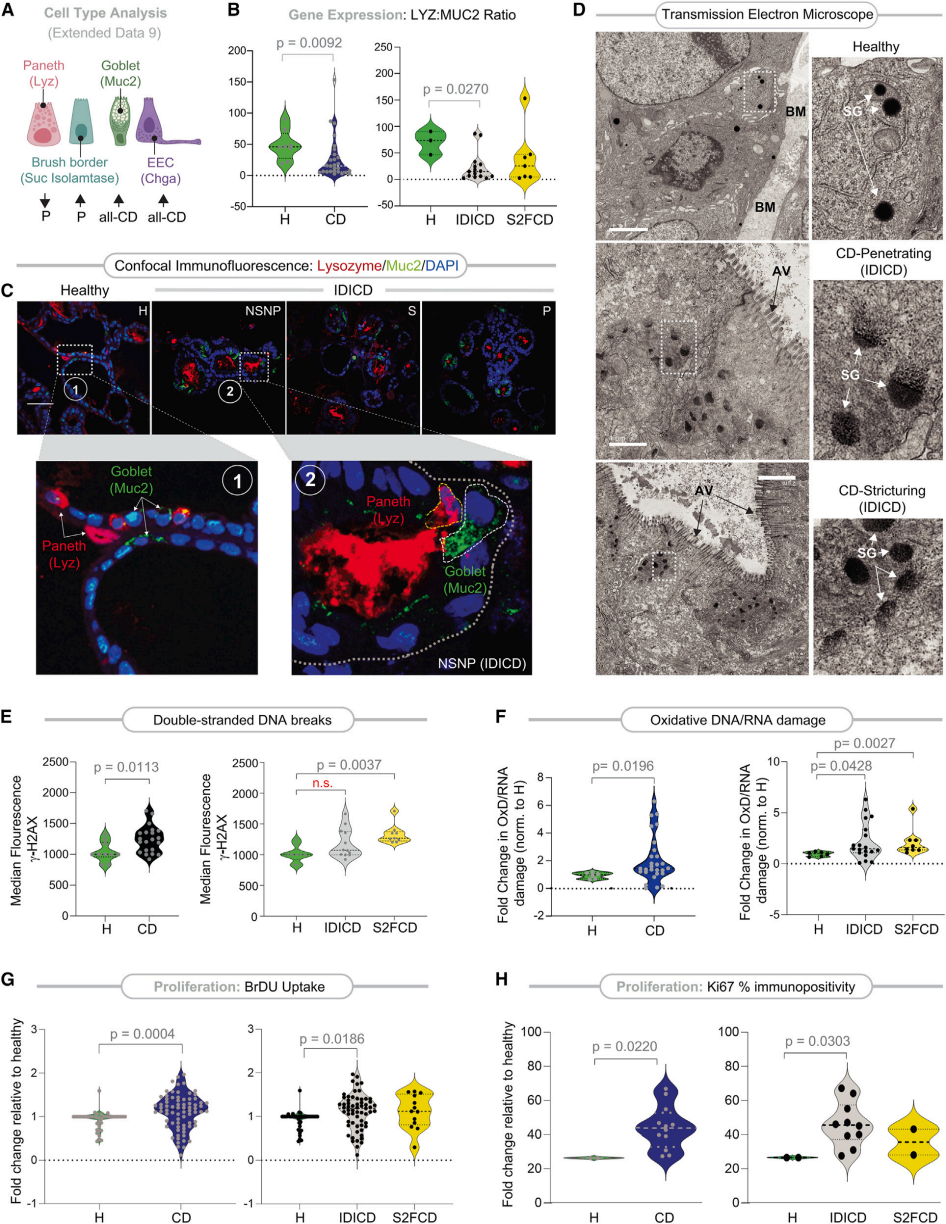
CD PDOs retain evidence of altered cell composition, high oxidative stress, and turnover (A) Schematic summarizing the relative expression of type, as determined by gene expression (markers used for each cell type in parentheses). Up-arrow, upregulation. Down-arrow, downregulation. P, penetrating CD. See Figures S9A–S9D for violin plots displayed as both clinical and molecular subtypes of CD. (B) Violin plots show the ratio of LYZ and MUC2 transcripts in CD PDOs vs. healthy controls (B [left], all CD subtypes combined; B [right], separated into molecular subtypes of CD). Statistical significance was assessed by Mann-Whitney. Only significant *p* values are displayed (*n* = 6–15 subjects in each group). See Figure S9E for the same data, displayed as clinical subtypes of CD. (C) FFPE of CD PDOs of the IDICD subtypes were analyzed for goblet (MUC2; green) and Paneth (lysozyme; red) cells by confocal immunofluorescence. Representative images are shown. Scale bar, 150 μm. Boxed regions (numbered 1, 2 in upper panels) are magnified below. See Figure S9F for quantification of images and Figure S9G for summary. (D) Electron micrographs of Paneth cells in healthy and CD PDOs of IDICD subtypes are shown. The boxed region on the left is magnified on the right. BM, basement membranes; AV, apical villi; SG, secretory granules. Scale bar, 1 μm in top and middle panels and 2 μm in the bottom panel. (E) Violin plots show the extent of DNA damage, as determined by flow cytometry analysis of γH2AX in CD PDOs vs. healthy controls (E [left], all CD subtypes combined; E [right], separated into molecular subtypes of CD). Statistical significance was assessed by Mann-Whitney (left) or one-way ANOVA (right). Only significant *p* values are displayed. (F) Violin plots show the extent of oxidative DNA/RNA damage in CD PDOs vs. healthy controls. Statistical significance was assessed by Mann-Whitney. Only significant *p* values are displayed. See Figures S10A and S10B for the display of the findings based on clinical subtypes of CD. (G) Violin plots show the extent of BrDU incorporation over 24 h on four-day-old CD PDOs grown in 96-well plates prior to assessment by ELISA. Statistical significance was assessed by Mann-Whitney (left) and one-way ANOVA (right). Only significant *p* values are displayed (*n* = 5–8 subjects in each group; 2–3 repeats in each PDO). See Figures S10C and S10D for the display of the findings based on clinical subtypes of CD. (H) Violin plots show % cells with Ki67-positive nuclei in CD PDOs vs. healthy controls. Statistical significance was assessed by Mann-Whitney. Only significant *p* values are displayed (*n* = 2–5 subjects in each group; 2–3 repeats in each PDO). See Figures S10E and S10F for the display of the findings based on clinical subtypes of CD. See Table S2 for subjects analyzed in each assay. See also Figure S11 for cellular apoptosis in CD PDOs at baseline and upon challenge with TNF-α.

We next assessed the CD PDOs for epithelial indicators of inflammation and stress, i.e., altered mucin production and cell composition^55^ and genotoxic stress.^56^ *MUC2* transcript, a marker of goblet cells, was elevated in CD PDOs, primarily in the IDICD PDOs, as determined by qPCR (Figures S9A and 4A). Although the transcripts of lysozyme (*LYZ*), a marker of the Paneth cells, were unchanged in CD PDOs (Figure S9B), we observed a reduced *LYZ*:*MUC2* ratio, indicative of a skewed ratio of secretory cells—Paneth and goblet cell—in CD PDOs, largely attributable to IDICD PDOs (Figure 4B). Confocal immunofluorescence studies showed reduced lysozyme-positive cells but an increased presence of luminal lysozyme and mucin-producing goblet cells in all CD PDOs (Figure 4C). Enhanced Paneth cell degranulation and granule depletion were confirmed at a higher resolution by TEM (Figure 4D). These observations are consistent with prior observations of enhanced extrusion and Paneth cell degranulation,^57^^,^^58^ increased thickness of mucin,^59^ and the number of goblet cells^60^ in CD. Because IFNγ induces Paneth cell degranulation,^57^^,^^58^ and because high serum IFNγ levels in CD originate from the immune cell infiltrates in the intestine,^61^ it was surprising that our CD PDOs retain this phenotype in culture despite being removed from those cells. Regardless, the findings agree with prior reports that, while healthy colons lack Paneth cells, colons from patients with IBD show metaplastic Paneth cells,^62^ which are a hallmark of IBD.^63^^,^^64^^,^^65^ Significant expansion of enteroendocrine cells was found in all CD PDOs across every clinical subtype (Figures S9C and 4A), as determined by the abundance of *CHGA* transcripts, which is consistent with prior reports of observed increases in CD tissues.^66^ Sucrase isomaltase, a marker of brush border enterocyte^67^ that is also found in terminally differentiated absorptive colonocyte,^68^ is increased in CD PDOs, specifically in the penetrating (P, B3) CD PDOs (Figures S9C and 4A).

Compared to healthy PDOs, CD PDOs showed increased genotoxic stress. Double-stranded DNA breaks (Figure 4E, *left*) and oxidative nucleotide damage (Figure 4F, *left*) were increased, as determined by γH2AX-based flow cytometry and in ELISA-based measurements of oxidized RNA/DNA products, respectively. S2FCD PDOs largely contributed to this increase (Figure 4E, *right*; 4F, *right*). As for clinical subtypes, increased genotoxic stress was primarily encountered in NSNP (B1) and more prominently in stricturing (S, B2) CD PDOs, whereas penetrating CD was relatively spared (Figures S10A and S10B).

That CD PDOs retain DNA damage, “Paneth cell degranulation,” and altered cellular composition phenotypes *ex vivo* suggests the retention of some epigenetic memory in the adult stem cell, likely imprinted by IFNγ, TNF-α, or other stressful triggers from the diseased tissue of origin.

#### IDICD shows higher cell proliferation and turnover

Because barrier loss due to increased death and/or impaired regeneration is a constant feature in inflammatory colitis,^69^ we next analyzed these properties in CD PDOs. Two complementary approaches were used—estimation of bromodeoxyuridine (5-bromo-2′-deoxyuridine [BrdU]) incorporation in colorimetric assays (Figure S10C) and staining formalin-fixed paraffin-embedded (FFPE) organoids for the Ki67 protein (MKI67; Figure S10E), a reliable marker of proliferation in the colon crypts.^70^ Both assays agreed, in that, CD PDOs display higher proliferation (Figures 4G, *left* and 4H, *left*); however, a subtype-specific analysis showed that increased proliferation was primarily and most consistently encountered in the IDICD subtype (Figures 4G, *right* and 4H, *right*). Among the clinical subtypes in IDICD, stricturing (S, B2) CD PDOs showed the most proliferation (Figures S10D and S10F).

When the same PDOs under similar conditions as earlier were assessed for apoptosis by TUNEL assays (Figure S11A), apoptosis was found to be increased at baseline in CD PDOs (all subtypes combined; Figure S11B, *left*). Subtype-specific analysis showed that apoptosis is more pronounced in the stricturing (S, B2) and penetrating (P, B3) subgroups (Figure S11B, *right*), but similar across the molecular subtypes (Figure S11C). When TUNEL assays were repeated with or without TNF-α challenge, apoptosis in response to such challenge was increased significantly only in NSNP (B1) and stricturing (S, B2) CD PDOs (Figure S11D). All 3 subtypes of CD PDOs showed increased apoptosis compared to TNF-α-challenged healthy PDOs (Figure S11D). Flow cytometry-based TUNEL analyses were attempted but were not interpretable because disruption of organoids was associated with artifacts.

The findings demonstrate that IDICD, but not S2FCD, shows a higher rate of apoptosis and proliferation and, hence, higher cell turnover (see Table 1), which agrees with prior observations in the CD colon.^69^^,^^71^^,^^72^^,^^73^ Because cytokine-induced apoptosis appears to be functionally far more relevant than spontaneous apoptosis in causing barrier dysfunction^74^ in IBD, results in TNF-α-challenged conditions indicate that the CD PDOs may have a wider range of defects in the milieu of inflammation.

### S2FCD and IDICD show subtype-defining phenomes that are therapeutically reversible

**Figure 5:**
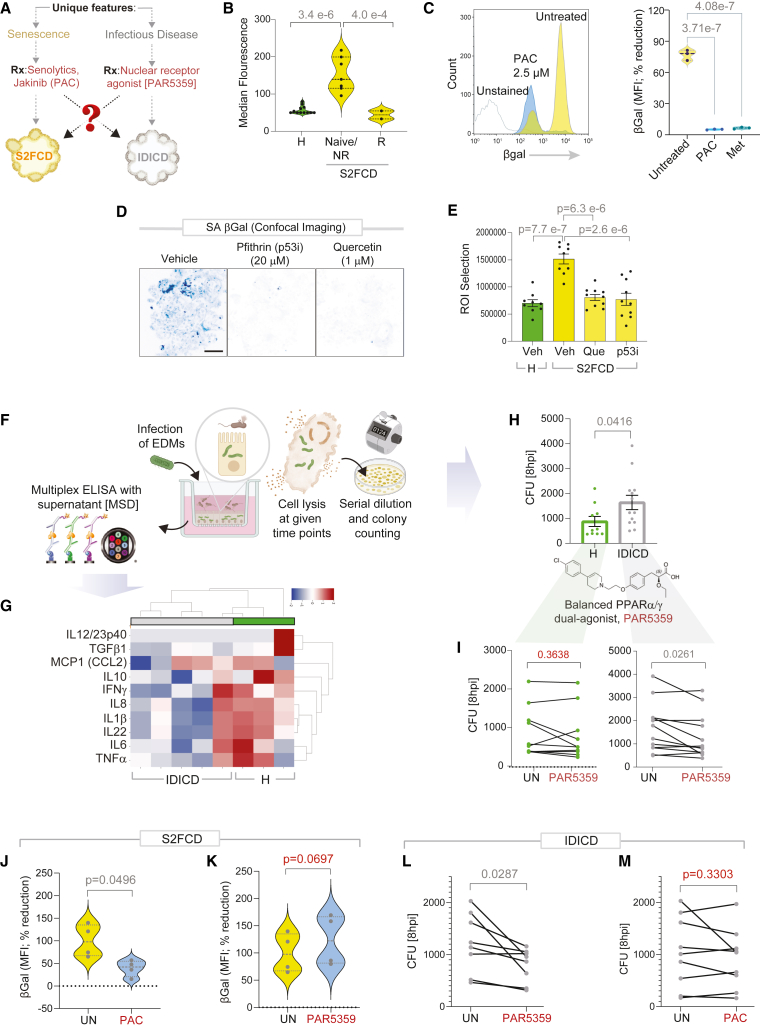
Genotyped-phenotyped CD PDOs can serve as platforms for personalized therapeutics (A) Schematic outlines the strategy for therapeutic reversal of the disease driver phenotypes in each molecular subtype of CD (B–I) and for crossover efficacy across the subtypes (J–M). (B) Mean fluorescent intensity of SA-βGal staining of PDOs by flow cytometry (B- yellow cluster CD PDOs separated into groups that responded (R) or not (NR) to anti-TNF-α biologics or were naive to that treatment). Data represents 2–4 technical repeats on 4 healthy and 6 CD PDOs. Statistical significance was assessed by one-way ANOVA. (C) Histogram (left) and violin plots (right) show the % changes in the median fluorescence intensity when CD PDOs were treated with senotherapeutics (2.5 μM PAC, pacritinib; 1 mM Met, metformin). Statistical significance was assessed by one-way ANOVA. (D–E) Inverted images displayed (D) are representative of ∼10 fields/sample of max-projected z stacks of CD PDOs stained with SPiDER-SA-β-Gal. Bar graphs (E) display the quantification of staining. Scale bar, 100 μm. Statistical significance was assessed by one-way ANOVA. (F) Schematic outlines the 3 major steps in bacterial clearance assays, and the concomitant assessment of supernatants for cytokines by multiplexed ELISA. (G) Heatmap displays the results of hierarchical agglomerative clustering of *AIEC*-LF82-challenged healthy and CD-EDMs using the cytokine profiles determined by mesoscale (MSD). See Table S7. (H) Bar plots show the abundance of bacteria retained within healthy (H) and IDICD EDMs at 8 h after infection. Statistical significance was assessed by t test. (I) Line plots show the pre-(UN) and post-treatment (Rx) effect of a balanced PPARα/g dual agonist (1 μM PAR5359) on the abundance of bacteria at the 8 h time point in healthy (left) and IDICD (right) EDMs. Statistical significance was assessed by t test. (J and K) Violin plots show the % change in median fluorescence intensity with PAC (J) or PAR5359 (K), as determined on S2FCD PDOs with the highest senescence in (B). Statistical significance was assessed by t test. (L and M) Line plots show the untreated and effect of PAR5359 (L) or PAC (M) treatment on the abundance of bacteria at the 8 h time point in IDICD EDMs with the highest bacterial load in H. Statistical significance was assessed by t test. See Table S2 for subjects analyzed in each assay.

Next, we asked if the predicted unique disease-defining phenotypes of CD PDOs can be detected in the PDOs (Figure 5A) so that they can be tracked and reversed by rationally paired therapeutics.

In the case of S2FCD, senescence was predicted as the unique disease-defining phenotype. We found that compared to healthy PDOs, the S2FCD PDOs displayed increased senescence in SA-βGal assays^75^; this phenotype was more pronounced in those who were biologic naive or non-responder to anti-TNF-α therapy (Figure 5B). Senescence was reversed by pacritinib (PAC; Figure 5C), which inhibits JAK1/2 kinases that are prominent drivers of cellular senescence and its profibrogenic senescence-associated secretory phenotype.^76^ Metformin, which reverses numerous hallmarks of senescence,^10^ was used as a positive control in these assays (Met; Figure 5C). Reduced senescence was also observed with the senolytic quercetin-3-D-galactose (Que) and the senomorphic pifithrin-α-p53 inhibitor (p53i) in the CD PDOs (Figures 5D and 5E).

In the case of IDICD, immune deficiency in the setting of infection was predicted as the unique disease-defining phenotype. We infected EDMs prepared using healthy and IDICD PDOs and analyzed them for their ability to mount a cytokine response and clear the infection (i.e., bacteria clearance assay; Figures 5F–5H). For mimicking infection, we chose the pathogenic adherent invasive *Escherichia coli* strain *LF82* (AIEC*-LF82*), which was isolated from CD patients,^77^ and applied it to the apical surfaces of polarized EDMs using well-established protocols.^22^^,^^51^ Cytokine response was assessed by carrying out ultrasensitive, ELISA-based high throughput (HTP) proteome studies on the supernatants collected from infected EDMs. Compared to healthy EDMs, IDICD EDMs showed impaired production of cytokines (Figure 5G). This defective cytokine response was only observed in the setting of infection (not at basal state without bacterial challenge) and only restricted to the IDICD PDOs, but not S2FCD PDOs (Table S7). Poor cytokine response in IDICD EDMs was also accompanied by impaired clearance (i.e., more live bacteria recovered at 8 h post-infection; Figure 5H), indicating that CD-EDMs fail to clear bacteria and/or permit more replication. These findings agree with the prior findings of downregulated transcripts of multiple cytokines (Figure 2D, *bottom left*) and confirm the presence of an immune-suppressed state in the setting of infection. We asked if impaired bacterial clearance in CD-EDMs can be reversed by a balanced and potent dual agonist of PPARα/γ, PAR5359,^78^ which has recently been shown to accelerate bacterial clearance in CD patient-derived PBMCs and ameliorate colitis in mice through the balanced induction of pro- and anti-inflammatory cytokines and ROS.^79^ We found that treatment of the CD-EDMs with PAR5359 significantly reduced the bacterial burden (Figure 5I, *right*), with virtually no effect on healthy EDMs (Figure 5I, *left*).

Although the phenotype of epithelial barrier dysfunction was not a prominent defect and was observed primarily in the NSNP (B1) CD PDOs, we asked if this too can be reversed. We prioritized two agents. The first is the probiotic drug *E coli* Nissle 1917 (*EcN;* Figure S12A), which is safe and effective in maintaining remission equivalent to the gold standard mesalazine in patients with UC, but with dubious results in CD.^80^^,^^81^ The second is the postbiotic Hylak Forte, which contains metabolic products (e.g., short-chain fatty acids, amino acids, and vitamins) derived from commensal microbes (Figure S12B). Both agents significantly increased the TEER in NSNP (B1) CD-EDMs, and in stricturing (B2) CD-EDMs.

### Subtype-personalized therapeutics lack crossover efficacy

The remarkable degree of internal consistency between the transcriptome, genome, and phenome led us to ask if the molecular subtypes represent an opportunity for personalized therapeutics. If so, the phenotype-based pairing of therapeutics in one molecular subtype should lack efficacy when crossed over to the other subtype (Figure 5A). We compared head-to-head the efficacy of the lead drug-like candidates—PAC and PAR5359—on the most diseased CD PDOs, i.e., those that were found to be the most senescent (in Figure 5B) or the most impaired in their ability to clear microbes (in Figure 5H). While PAC was effective in reversing senescence in S2FCD PDOs (Figure 5J), PAR5359 was not (Figure 5K). Similarly, while PAR5359 was effective in improving microbial clearance in IDICD PDOs (Figure 5L), PAC was not (Figure 5M). The findings suggest that subtype-specific therapeutic pairing may improve precision in targeting the disease-driving features within each subtype.

### Conclusion

The major discovery we report here is the identification of two distinct molecular subtypes of CD—IDICD and S2FCD—based exclusively on the properties of the epithelial stem cells in the colon. Although PDOs have previously been shown to faithfully retain the tissue phenotypes in both CD ileum^6^ and in UC colon,^7^ our findings reveal the surprising potential for genotyped-phenotyped CD colon-derived PDOs as platforms for implementing personalized medicine. They also represent a paradigm shift in how we classify CD from clinical patterns to dysregulated molecular pathways that directly suggest therapeutic interventions. There are three major impacts of these findings.

#### A CD molecular classification that reconciles genome, transcriptome, and phenome

Finding a robust internal consistency between genome, transcriptome, proteome (cytokines), and phenome that overcame the heterogeneity of clinical presentation and cohort composition is striking. The IDICD subtype shows impaired pathogen clearance and insufficient cytokine response. This subtype frequently harbors the major CD risk alleles of NOD2 and ATG16L1 and shows skewed differentiation in the PDOs. The S2FCD subtype is characterized by cellular senescence and genotoxic stress and displays a profibrotic transcriptome. The genome of this CD subtype shows higher mutations in the YAP1-IL-18 pathway, which has recently been implicated in telomere instability-associated tissue inflammation that originates in the CD colonic epithelium.^43^ These findings are consistent with prior work showing that DNA damage serves as a trigger for senescence and inflammation in CD.^82^ Up until now, the contributions of genetics as a determinant in the various clinical behaviors of CD have remained unclear^3^; the alignment of genetics with the transcriptome and phenome within each molecular subtype suggests that the molecular subtypes (revealed here) cannot be explained simply on the basis of genomic alterations but also reflect the altered epigenome. Having both represented could be a more meaningful way to classify patients because such classification is linked to driver phenotypes of the disease with therapeutic implications. Finally, that perianal disease mimics penetrating (B3) CD at a fundamental molecular level is another important insight, which should be exploited in determining management strategies for these patients.

#### Molecular classification that informs the choice of therapeutics

Efforts at the successful development of CD therapeutics have stumbled because we lack understanding of what drives CD heterogeneity and evolution. That a vast majority of the patients belong to the IDICD molecular subtype may explain the observed futility^17^ of the currently FDA-approved immunosuppressive drugs as agents to maintain remission in the B3-penetrating clinical subtype (which, belong to the IDICD molecular subtype). Our proof-of-concept studies also reveal that CD PDOs could be used to directly test drug efficacy in a personalized treatment approach; PDOs could be classified into one of the two major molecular subtypes and subsequently tested for therapeutic efficacy using novel or clinically approved drugs within weeks after derivation. In doing so, CD PDOs may fill the gap between imperfect cell/animal models and limitations of CD GWASs and clinical trials and allow personalized therapy selection. For example, the pan-JAK-inhibitor tofacitinib has succeeded in phase 3 trials for UC, with conflicting results in CD,^83^ and our findings suggest that targeted trials on the S2FCD molecular subtype using JAK1/2-selective inhibitors (e.g., upadacitinib, filgotinib, or baricitinib) may have demonstrable efficacy. As for IDICD, the strategy of improving microbial clearance using the balanced dual-PPARα/γ agonism with PAR5359 was identified using an AI-guided network transcriptomics approach,^20^^,^^79^ which is predicted to protect the gut mucosal barrier in IBD and represents a new class of therapy in IBD yet to enter clinical trials.

#### A blueprint for benchmarking PDOs as models to spur drug discovery

Recapitulating the active diseased epithelium (and its exposome), despite being removed for prolonged duration (∼weeks to months) from the dysbiotic lumen and the inflamed *in vivo* conditions, suggests that our adult stem cell-derived PDOs retained sufficient “memory” of the disease state. We confirmed this by objective benchmarking metrics, i.e., gene expression in PDOs vs. CD epithelium (Figures 2C and S1), not just pathway analyses, which lack reproducibility as annotations are updated. We attribute such faithful recapitulation to growth conditions that have a proven track record of reproducibility between laboratories^23^ and to the fact that we used colon-crypt-derived adult stem cells (instead of reprogrammed iPS cells). This disadvantage of iPS-derived organoids as models for IBD was predicted intuitively,^84^ but remained unproven until now.

### Limitations of the study

A side-by-side comparison of PDO vs. its tissue of origin or scSeq studies were not attempted. These concerns are somewhat mitigated by demonstrated similarities in cell type composition and >90% similarities in protein-coding genes between CD tissue and CD PDOs^8^ and through benchmarking of our PDOs using scSeq-derived insights from primary epithelial cells from CD colons.^10^ Although we suggest that epigenetic events are likely contributors that preserve the memory of the *in vivo* diseased state in the PDOs, the nature of these events was not explored here. Although we prioritized the colon because it is involved in 60% of patients with CD,^18^ if the colon is a determinant or site of origin of ileal disease was not studied; further studies with matched acquisitions from same subjects will pave the way to such investigations. We also did not assess the ileum or the microbiome; such studies are expected to reveal if the nature of dysbiosis is unique to the molecular subtypes. While this study focused on targeted genomics to recapitulate the current practices for clinical research and trial design, whole-genome and/or whole-exome sequencing studies are required to fully evaluate the degree of convergence between the genome and the phenome.

## Resource availability

### Lead contact

Further information and requests for resources and reagents should be directed to and will be fulfilled by the lead contact, Pradipta Ghosh, prghosh@ucsd.edu.

### Materials availability

This study has generated an organoid biobank, RNA and DNA from the organoids. These materials are available from the lead contact with a completed Materials Transfer Agreement and patented technology (Das and Ghosh) agreement following the guidelines of the University of California, San Diego.

### Data and code availability

• Newly generated transcriptomic datasets reported in this paper have been deposited in NCBI’s Gene Expression Omnibus. Processed scRNA sequencing files are accessible through the GEO series accession number GEO: GSE192819. Publicly available datasets are accessible through the GEO series accession numbers GEO: GSE16879, GSE115390, and E-MTAB-7604.
• All original code has been deposited at Zenodo at [https://doi.org/10.5281/zenodo.13775972] and is publicly available as of the date of publication.
• Any additional information required to reanalyze the data reported in this work paper is available from the lead contact upon request.

## Acknowledgments

This work was supported by 10.13039/100007028The Leona M. and Harry B. Helmsley Charitable Trust (to P.G. and S.D.). Other sources of support include 10.13039/100000002National Institutes of Health (NIH) grants AI141630 (to P.G.), DK107585, R56 AG069689, and DiaComp Pilot and Feasibility award (to S.D.) and R01-GM138385 and Padres Pedal the Cause/C3 Collaborative Translational Cancer Research Award (San Diego NCI Cancer Centers Council [C3] #PTC2017) (to D.S.). P.G., S.D., and D.S. were also supported by 10.13039/100000002NIH awards UG3TR003355, UH3TR003355, UG3TR002968, and R01-AI155696. B.S.B. was supported by 10.13039/100000002NIH awards K23DK123406 and P30DK120515. G.D.K. was supported through The American Association of Immunologists Intersect Fellowship Program for Computational Scientists and Immunologists. S.-R.I. was supported by the postdoctoral fellowship grant from 10.13039/100000002NIH (3R01DK107585-02S1). J.E. was supported by an NCI/NIH-funded Cancer Biology, Informatics & Omics (CBIO) Training Program (T32 CA067754) and a Postdoctoral Fellowship from the 10.13039/100000048American Cancer Society (PF-18-101-01-CSM). This publication includes data generated at the UC San Diego IGM Genomics Center utilizing an Illumina NovaSeq 6000 that was purchased with funding from a National Institutes of Health SIG grant (#S10 OD026929). We are grateful to Helen Le and Jennifer Neill (UCSD IBD Center), Donald Pizzo (UCSD Pathology Histologic Biomarkers Core), and Ying Jones (UC San Diego Electron Microscopy Core Facility) for technical and logistical support.

## Author contributions

S.D. and P.G. conceptualized, supervised, and administered the project and acquired funding to support it. C.T., A.G.F., G.D.K., I.M.S., S.-R.I., R.F.P., M.F., P.M., D.L.S., S.D., and P.G. were involved in organoid isolation, culture, their use in various experiments, data curation, and formal analysis. Computational analyses were carried out by S.T. under the supervision of P.G. and D.S. D.S. provided all computational software. H.N.L., W.J.S., and B.S.B. provided key resources for human subjects and were responsible for the selection and enrolling of patients into this study. C.T., A.G.F., S.D., and P.G. prepared figures for data visualization and wrote the original draft. C.T., A.G.F., B.S.B., D.S., S.D., P.G., I.M.S., and G.D.K. reviewed and edited the draft. All co-authors approved the final version of the manuscript.

## Declaration of interests

S.D. and P.G. have a patent on the methodology.

## STAR★Methods

### Key resources table

**Table:** 

REAGENT or RESOURCE	SOURCE	IDENTIFIER
**Antibodies**		

Recombinant Anti-Collagen I Antibody	Abcam	Cat# ab270993; RRID:AB_2927551
Anti-β-catenin Antibody (E−5)	Santa Cruz	sc-7963; RRID:AB_626807
E-cadherin Antibody (H-108)	Santa Cruz	sc-7870; RRID:AB_2076666
Recombinant Anti-Ki67 antibody [SP6]	Abcam	ab16667; RRID:AB_302459
P-Histone H2A.X (Ser 139)	Santa Cruz	sc-517348; RRID:AB_2783871
Lysozyme Polyclonal Antibody	Invitrogen	PA5-16668; RRID:AB_10984852
MUC2 Monoclonal Antibody (996/1)	Invitrogen	MA5-12345; RRID:AB_10975230
Occludin Monoclonal Antibody (OC-3F10)	Invitrogen	33–1500; RRID:AB_2533101
Vimentin Monoclonal Antibody (3H9D1)	Proteintech	60330-1-Ig; RRID:AB_2881439
ZO-1 antibody [N2C1], Internal	GeneTex	GTX108627 RRID:AB_10731582
Goat Anti-Rabbit Alexa Fluor 488 Antibody	Abcam	Cat# 150077 RRID: AB_2630356
Goat Anti-Mouse Alexa Fluor 488 Antibody	Invitrogen	A11001; RRID:AB_2534069
Goat Anti-Rabbit Alexa Fluor 594 Antibody	Invitrogen	A11012; RRID:AB_2534079
Goat Anti-Rabbit Alexa Fluor 488 Antibody	Abcam	Cat# 150077 RRID: AB_2630356

**Bacterial strains**		

Adherent-Invasive *E. coli* LF82	Arlette Darfeuille-Michaud	N/A

**Chemicals, peptides, and recombinant proteins**		

MEM	Corning	Cat# MT 10-010-CV
Collagenase Type I	Thermo Fisher	Cat# 17100017
Collagenase Type II	Thermo Fisher	Cat# 17101015
Collagenase Type IV	Thermo Fisher	Cat# 17104019
HBSS with Ca2+/Mg2+	Thermo Fisher	Cat# 14-025-092
HBSS without Ca2+/Mg2+	Thermo Fisher	Cat# 14-175-095
MEM Non-Essential Amino Acids	Thermo Fisher	Cat# 11-140-050
Intestigro™ [L-WRN conditioned media, for intestine and colon PDOs]	UC San Diego HUMANOID™ Center	Cat# HUM2019
Tailor-2-Gro™ [L-WRN conditioned media, base]		Cat# HUM202420
Sodium Pyruvate	Sigma-Aldrich	Cat# S8636
Ciprofloxacin Hydrochloride	Corning	Cat# 61-277-RF
Antibiotic Antimycotic	Sigma-Aldrich	Cat# A5955
Trypsin (2.5%)	Thermo Fisher	Cat# 15090046
Zinc Formalin	Fisher Scientific	Cat# 23-313096
Xylene	VWR	Cat# XX0060-4
Hematoxylin	Sigma-Aldrich Inc	Cat# MHS1
Ethanol	Koptec	Cat# UN1170
Sodium Citrate	Sigma-Aldrich	Cat# W302600
DAB (10x)	Thermo Fisher	Cat# 1855920
Stable Peroxidase substrate buffer (10x)	Thermo Fisher	Cat# 34062
3% Hydrogen Peroxide	Target	Cat# 245-07-3628
Horse Serum	Vector Labs	Cat# 30022
Paraformaldehyde 16% Solution, EM Grade	Electron Microscopy Sciences	Cat# 15710
100% Methanol	Supelco	Cat# MX0485
Glycine	Fisher Scientific	Cat# BP381-5
Bovine Serum Albumin	Sigma-Aldrich	Cat# A9647-100G
Triton X-100	Sigma-Aldrich	Cat# X100-500ML
Prolong Glass	Invitrogen	Cat# P36984
Nail Polish (Rapid Dry)	Electron Microscopy Sciences	Cat# 72180
Gill Modified Hematoxylin (Solution II)	Millipore Sigma	Cat# 65066-85
Histogel	Thermo Scientific	Cat# HG4000012
TrypLE Select	Thermo Scientific	Cat# 12563-011
Advanced DMEM/F-12	Thermo Scientific	Cat# 12634-010
HEPES Buffer	Life Technologies	Cat# 15630080
Glutamax	Thermo Scientific	Cat# 35050-061
Penicillin-Streptomycin	Thermo Scientific	Cat# 15140-122
Matrigel	Corning	Cat# 354234
DPBS	Thermo Scientific	Cat# 14190-144
Ultrapure Water	Invitrogen	Cat# 10977-015
EDTA	Thermo Scientific	Cat# AM9260G
Fetal Bovine Serum	Sigma-Aldrich	Cat# F2442-500ML
Cell Recovery Solution	Corning	Cat# 354253
Sodium Azide	Fisher Scientific	Cat# S227I-100
Cyto-Fast Fix/Perm Buffer Set	BioLegend	Cat# 426803
FITC-Dextran	Sigma-Aldrich	Cat# FD10S
Ethyl alcohol, pure	Sigma-Aldrich	Cat# E7023
TRI Reagent	Zymo Research	Cat# R2050-1-200
Metformin	Sigma	Cat# D150959-5G
Pacritinib	Selleck Chem	Cat# S8057
Quercetin	Sigma Aldrich	Cat# Q4951
TP53 Inhibitor (p53i; Pifithrin-α-HBr)	Adipogen Life Science®	Cat# AG-CR1-0004-M005
Hylak Forte™	Amazon.com	N/A

**Critical commercial assays**		

BrdU Cell Proliferation ELISA Kit (colorimetric)	Abcam	Cat# ab126556
DNA/RNA oxidative damage ELISA kit	Cayman Chemical, USA	Cat # 589320
SPiDER-βGal	Dojindo	Cat# SG02-10
TUNEL Assay Kit - BrdU-Red	Abcam	Cat# ab66110
Quick-RNA MicroPrep Kit	Zymo Research	Cat# R1051
Quick-RNA MiniPrep Kit	Zymo Research	Cat# R1054
HRP Horse Anti-Rabbit IgG Polymer Detection Kit	Vector Laboratories	Cat# MP-7401

**Oligonucleotides**		

2x SYBR Green qPCR Master Mix	Bimake	Cat# B21203
qScript cDNA SuperMix	Quanta Biosciences	Cat# 95048

**Deposited data**		

RNA sequencing data of PDOs from the colons of patients with CD and UC (or healthy controls)	This paper	GEO: GSE192819
RNA sequencing data of intestinal mucosal biopsies colon from IBD patients with infliximab treatment	Arijs et al.^85^	GEO: GSE16879
RNA sequencing data from CD patients	Corraliza et al.^86^	GEO: GSE115390
RNA sequencing data of inflamed intestinal mucosa of inflammatory bowel disease patients	Verstockt et al.^87^	E-MTAB-7604

**Software and algorithms**		

ImageJ	ImageJ	RRID:SCR_003070
GraphPad Prism	GraphPad Prism	RRID:SCR_002798
MSD® DISCOVERY WORKBENCH 4.0	MSD	N/A
LAS AF Software	LAS AF Software	N/A
QuantStudio Design & Analysis Software	QuantStudio Design & Analysis Software	N/A
CIBERSORTx	CIBERSORTx	N/A
FlowJo	Flow Jo V10, BD BioSciences	RRID:SCR_008520

**Other**		

6-well Tissue Culture Plate	Genesee Scientific	Cat# 25-105
12-well Tissue Culture Plate	CytoOne	Cat# CC7682-7512
Transwell Inserts (6.5 mm, 0.4 μm pore size)	Corning	Cat# 3470
Cell Scraper	Millipore Sigma	Cat# C5981-100EA
Millicell EZ Slide 8-Well Chamber	Millipore Sigma	Cat# PEZGS0816
Trypan Blue Stain	Invitrogen	Cat# T10282
70 μm Cell Strainer	Thermo Fisher Scientific	Cat# 22-363-548
Noyes Spring Scissors - Angled	Fine Science Tools	Cat# 15013-12
gentleMACS™ C Tubes	Miltenyi Biotec	Cat# 130-093-237
DAPI	Invitrogen	Cat# D1306 RRID: AB_2629482
Phalloidin, Alexa Fluor 594	Invitrogen	Cat# A12381 RRID: AB_2315633
Propidium Iodide	Invitrogen	Cat# V-13245 B
Countess II Automated Cell Counter	Thermo Fisher Scientific	AMQAX1000
Epithelial Volt-Ohm (TEER) Meter	Millipore	MERS00002
Automated TEER measurement system [REMS AutoSampler]	World Precision Instruments (WPI)	N/A
MESO QuickPlex SQ 120	MSD	N/A
Leica TCS SPE Confocal	Leica Microsystems	TCS SPE
Power Pressure Cooker XL	Tristar Products	N/A
Canon Rebel XS DLSR	Canon	N/A
MiniAmp Plus Thermal Cycler	Applied Biosystems	Cat# A37835
QuantStudio5	Applied Biosystems	Cat# A28140 RRID:SCR_020240
Light Microscope (brightfield images)	Carl Zeiss LLC	Axio Observer, Inverted; 491917-0001-000
Fisherbrand™ 150 Handheld Homogenizer	Fisher Scientific	Cat# 15340168
Spark 20M Multimode Microplate Reader	Tecan	N/A
NanoQuant Infinite M200	Tecan	N/A
Guava® easyCyte Benchtop Flow Cytometer	Millipore	Guava easyCyte 6 2L
MESO QuickPlex instrument	Mesoscale Discovery Inc.	SQ 120
gentleMACS™ Dissociator	Miltenyi Biotec	Cat# 130-093-235

### Experimental model and subject details

#### Human subjects

For generating healthy and CD patient-derived organoids (PDOs), patients were enrolled for colonoscopy as part of routine care for the management of their disease from the University of California, San Diego IBD-Center, following a research protocol compliant with the Human Research Protection Program (HRPP) and approved by the Institutional Review Board (Project ID# 1132632: PI Boland and Sandborn). Histologically normal healthy colon samples were collected from patients presenting for screening colonoscopy or undergoing the procedure for making the diagnosis of irritable bowel syndrome. Each participant provided a signed informed consent to allow for the collection of colonic tissue biopsies for research purposes to generate 3D organoids. Isolation and biobanking of organoids from these colonic biopsies were carried out using an approved IRB (Project ID # 190105: PI Ghosh and Das) that covers human subject research at the UC San Diego HUMANOID Center of Research Excellence (CoRE). For all the deidentified human subjects, information including age, gender, and previous history of the disease, was collected from the chart following the rules of HIPAA. The study design and the use of human study participants was conducted in accordance to the criteria set by the Declaration of Helsinki.

#### Isolation of enteroids from colonic specimens of healthy and Crohn’s disease subjects

Intestinal crypts, comprised of crypt-base columnar (CBC) cells, were isolated from human colonic tissue specimens using the previously published paper.^20^^,^^21^^,^^22^^,^^52^ In brief, intestinal crypts were dissociated from tissues by digesting with collagenase type I (2 mg/mL solution containing gentamicin 50 μg/mL). The plate was incubated in a CO_2_ incubator at 37°C, mixing every 10 min with vigorous pipetting in-between incubations, while monitoring the release of single epithelial units from tissue structures by light microscopy. To inactivate collagenase, wash media (DMEM/F12 with HEPES, 10% FBS) was added to cells, filtered through a 70 μm cell strainer, centrifuged at 200 g for 5 min and then the supernatant was aspirated, leaving behind a cell pellet. The number of viable intestinal stem cells was determined by the Trypan Blue Exclusion method using Countess II Automated Cell Counter. Epithelial units were resuspended in Matrigel and 25 μL of cell-matrigel suspension was added to the wells of a 12-well plate on ice and incubated upside-down in a 37°C CO_2_ incubator for 10 min, which allowed for polymerization of the Matrigel. After 10 min of incubation, 1000 μL of 50% conditioned media (purchased from the UC San Diego HUMANOID Center; Intestigro; Cat#HUM2019), prepared from L-WRN cells^19^ [ATCC CRL-3276] with Wnt3a, R-Spondin and supplemented with 20 ng/mL EGF, 10 μM SB202190 (p38 MAPK inhibitor), 10 μM Y27632 (Rho-associated, coiled-coil containing protein kinase (ROCK) inhibitor) and 10 μM SB431542 (TGFβ/SMAD inhibitor). The medium was changed every 2 days and the enteroids were either expanded or frozen in liquid nitrogen for biobanking. The number of PDOs varied between patient samples, with some tissues rendering hundreds-to-thousands of organoids, whereas others yielded only tens-to-hundreds primary organoids. Organoids from patients with active inflammation took longer to establish than those without inflammation. All experiments were conducted using passages <10; and in most instances, the phenotypes held true up to passage 15. No studies were conducted above passage 15.

#### Preparation of enteroid-derived monolayers (EDMs)

EDMs were prepared by dissociating single cells from enteroids and plated either in 24-well or 96-well transwell with a 0.4 μm pore polyester membrane coated with diluted Matrigel (1:40) in 5% conditioned media as done before.^20^^,^^21^^,^^22^^,^^52^ The single-cell suspension was seeded at a density of approximately 2x10^5^ cells/well (in case of 24-well) or 8x10^4^ cells/well (in case of 96-well) and EDMs were differentiated for 2–3 days in 5% conditioned media, which is prepared by diluting Intestigro with Tailor-2-Gro (HUMANOID Center; cat# HUM202402). The media was changed every 24 h and monitored under a light microscope to evaluate the EDM generation and quality. As expected, the expression of EDMs showed a significant reduction of the stemness marker *LGR5* in EDMs.^21^^,^^25^^,^^52^

### Method details

#### Experimental methods

##### Quantitative assessment of organoid morphology by Imaris

.LIF files were first converted into native IMARIS format (.ims). Then a spots filter and surface filter were created. This filter is used as a batch function on all processed images. Finally, a cell object is created where broken fragments of single organoids are stitched together manually. Upon manually completion specific measurements are exported from IMARIS to GraphPad Prism for further analysis and for visualization as graphs.

##### TUNEL assay

The baseline level and the effect of TNFα on apoptosis between healthy and CD organoids was quantified using the TUNEL Assay Kit via immunofluorescence (see key resources table). Organoids were seeded at a density of 5000 cells/well on a layer of Matrigel in an 8-well chamber slide and cultured for 4 days in growth media. TNFα (100 ng/mL) treatment was applied for 24 h prior to fixation (day 3). Organoids were fixed in 4% paraformaldehyde (PFA) at room temperature for 30 min and quenched with 30 mM glycine for 5 min. Subsequently, samples were processed as per manufacturer’s specification, i.e., they were permeabilized and blocked for 1 h using blocking buffer (2 mg/mL BSA and 0.1% Triton X-100 in PBS), washed with PBS and then treated with 100 μL of DNA labeling solution (diluted 1:1) in ultrapure water, and incubated subsequently in a dark humidified incubator for 1 h at 37°C. A negative control DNA labeling solution, which substituted the TdT labeling enzyme for ultrapure water, was used to determine assay background and noise. Samples were washed in PBS, treated with 100 μL of antibody solution (constituted according to the manufacturer’s specifications) supplemented with DAPI (1:500 dilution) and then incubated in the dark for 30 min at room temperature. Samples were washed in ultrapure water, mounted using Prolong Glass prior to application of coverslips (no. 1 thickness) and sealed. Slides were imaged immediately by confocal microscopy within 6 h of staining as per manufacturers recommendation.

##### Quantification of TUNEL assays

z stack images were acquired by successive 3 μm depth Z-slices of organoids using confocal microscopy. Maximum intensity projection of the stack of images were analyzed using the FIJI (ImageJ) Plugin, ‘RGB Measure’ to quantify the integrated density of the BrdU signal (red/594 nm channel). Total area of enteroids in a field was determined by converting DAPI (blue) channel to a binary image and thresholded to determine nuclear boundaries. The level of apoptosis was quantified by comparing apoptotic-positive cell signal (Brdu-Red) relative to the area of DAPI-positive cell signal. A blank subtraction, using the negative control, was done to normalize values across independent experiments. The effect of TNFα on each patient-derived organoid line was determined by fold change relative to its untreated sample counterpart. All images were processed on ImageJ software (NIH) and assembled into figure panels using Photoshop and Illustrator (Adobe Creative Cloud).

##### Assessment of cell proliferation by an ELISA-based BrdU incorporation assay

The level of proliferation was quantified with a BrdU Cell Proliferation ELISA kit (see key resources table). Organoids were seeded at a density of 3500 cells/well on a layer of Matrigel in 96-Well plate and cultured for 3 days in growth media. BrdU (1x) was added and allowed to incorporate into the organoids for 24 h prior to fixation on day 4. Cells were fixed according to the manufacturer’s instructions. Optical Density (OD) was measured using a 450 nm spectrophotometer microplate reader. Each patient cell line had negative BrdU control wells for the determination of background signal. The average negative BrdU control OD was subtracted from a patients average BrdU-incorporated OD to obtain a normalized OD value. Fold change was determined relative to a single healthy PDO, kept constant through all assays, for all samples including other healthy PDOs.

##### Assessment of cell proliferation by Ki67 particle analysis

z stack images were acquired by successive 3 μm depth Z-slices of Ki67 stained organoids using confocal microscopy. From the acquired maximum intensity projection images, the DAPI channel was converted to a grayscale image. A Gaussian blur filter of σ (Radius): 1 was applied to reduce nuclear noise. Manual thresholding was applied to convert grayscale image into a binary: black and white image; using the FIJI Default settings and Over/Under against a dark background. Overlapping particles were separated using the watershed method and particles were analyzed with the requirements; size: 5-infinity μˆ2, circularity: 0.4–1.0 and edge particles excluded. The auto generated DAPI particle outline was used as a boundary to manually count particles that contained positive Ki67 signal. The #Ki67 positive cells/total # of cells was used to generate the % of Ki67 positive cells. Approximately 10 random fields, that were representative of the overall staining observed in the samples were imaged per PDO.

##### Measurement of oxidative DNA/RNA damage

The amount of oxidative DNA damage in healthy and CD EDMs was quantified using a commercial kit (see key resources table) according to the manufacturer’s instructions and previously published papers by others^88^^,^^89^^,^^90^ and us.^91^^,^^92^ Briefly, supernatant from EDMs was used to detect oxidized guanine species: 8-hydroxy-2′-deoxaguanosine from DNA, and 8-hydroxyguanine from either DNA or RNA.

##### Estimation of Paneth:Goblet cell ratio by confocal imaging of cell markers

Fluorescent z stack images of lysozyme (a *bona-fide* marker of Paneth cells) and muc2 (a *bona-fide* marker of goblet cell) stained organoids were acquired by successive 1 μm depth Z-slices of EDMs in the desired confocal channels of Leica TCS SP5 Confocal Microscope as done previously.^52^ Fields of view that were representative of a given transwell were determined by randomly imaging 3 different fields. Z-slices of a z stack were overlaid to create maximum intensity projection images; all images were processed using FIJI (ImageJ) software. All images were processed on ImageJ software (NIH) and assembled into figure panels using Photoshop and Illustrator (Adobe Creative Cloud).

##### Measurement of LYZ: MUC2 ratio

From the acquired maximum intensity projection images both lysozyme (Red/594 nm channel) and MUC2 (green/488 nm channel) were converted to grayscale images. Particles were quantified, with the following thresholds-- size: 0-infinity μ^2^, circularity: 0.0–1.0 and edge particles were included to independently determine the total area positive for lysozyme and MUC2 signals. DAPI particle analysis was carried out the same as done for Ki67 particle analysis. Total area of cell-specific marker/total # of cells was used to generate a normalized value of area per cell for each marker, lysozyme and MUC2. Approximately 5–10 random fields that were representative of the staining were imaged per PDO sample. All images were processed on ImageJ software (NIH) and assembled into figure panels using Photoshop and Illustrator (Adobe Creative Cloud).

##### Embedding of organoids in HistoGel

Healthy and CD colonic organoids were embedded in histogel as done previously.^93^ Briefly, mature organoids after 7-dasys of culture in 6-Well plates were fixed in 4% PFA at room temperature for 30 min and quenched with 30 mM glycine for 5 min. After washing with PBS, organoids were resuspended in PBS and stained using Gill’s hematoxylin for 5 min for ease during embedding in paraffin blocks and visualization during and after sectioning. Excess hematoxylin was removed, and organoids were resuspended in HistoGel and centrifuged at 65°C for 5 min. HistoGel embedded organoid pellets were cooled to room temperature and stored in 70% ethanol at 4°C until ready for embedding in paraffin blocks. FFP-embedded organoid sections were cut at a setting of 4 μm thickness and fixed on to microscope slides for H&E staining.

##### Immunofluorescence of FFPE organoids

Sections of FFP-embedded healthy- and CD- PDOs were deparaffinized, rehydrated and underwent antigen retrieval immersed in Sodium Citrate buffer (pH 6.0) and boiled at 100°C inside a pressure cooker for 3 min. Once sections returned to room temperature, samples were washed in DI water and then permeabilized and blocked for 2 h using an in-house blocking buffer (2 mg/mL BSA and 0.1% Triton X-100 in PBS), as described previously.^93^^,^^94^ Primary antibodies [see key resources table] were diluted in blocking buffer and incubated overnight at 4°C. Secondary antibodies were diluted in blocking buffer and allowed to incubate for 2 h in the dark. Antibody dilutions are listed in the key resources table. ProLong Glass was used as a mounting medium. Coverslips (No.1 thickness) were applied to slides to seal and stored at 4°C until imaged.

##### Ultrastructural analyses of patient-derived organoids by electron microscopy

Organoid pellets were fixed with 2% Glutaraldehyde in 0.10 M cacodylate buffer and further postfixed in 1% OsO4 in 0.10 M cacodylate buffer for 1 h on ice. Organoids were stained with 2% uranyl acetate for 1 h on ice, following which they were dehydrated in a graded series of ethanol (50–100%) while remaining on ice. Organoids were then subjected to 1 wash with 100% ethanol and 2 washes with acetone (10 min each) and embedded with Durcupan. Sections were cut at 60 nm on a Leica UCT ultramicrotome and picked up on 300 mesh copper grids; different planes were obtained from consecutive 10 μm cuts. Sections were post-stained with 2% uranyl acetate for 5 min and Sato’s lead stain for 1 min. Images were acquired using a JEOL 1400 plus microscope equipped with a bottom-mount Gatan OneView (4k x 4k) camera.

##### Immunofluorescence imaging of epithelial tight junctions in EDMs

The media from apical and basolateral compartments of all EDMs were removed, washed 3 times with room temperature PBS, fixed with ice-cold 100% methanol at −20°C for 20 min. Afterward, methanol was removed and washed with blocking buffer (0.1% Triton TX-100, 2 mg/mL BSA diluted in PBS) to permeabilize EDMs and to incubate with the following primary antibodies overnight: ZO-1 (1:500) and Occludin (1:500). Primary antibodies were removed and washed with PBS 3 times for 5 min each time; after which the following secondary antibodies were added for 2 h: Alexa Fluor 594 conjugated goat anti-rabbit IgG, Alexa Fluor 488 conjugated goat anti-mouse IgG and DAPI. Secondary antibodies were removed and washed with PBS 3 times for 5 min each time. To preserve fluorescence, monolayers were treated with Prolong Gold antifade reagent and stored at 4°C until imaged. A confocal Microscope (Leica SPE) with a 40× objective lens was used to image the stained EDMs.

##### Measurement of trans-epithelial electrical resistance (TEER)

Two different methods in low- (LTP) and high-throughput (HTP) modes were used for the measurement of TEER as described before with some modifications.^22^ LTP assessment of TEER was carried out manually in 24-well transwell plates. TEER was measured at 24 h, and 48 h, following monolayer seeding using STX2 electrodes with digital readout by EVOM2 (WPI). HTP assessment of TEER was carried out in an automated manner in 96-well transwell plates. TEER was measured using the REMS AutoSampler (WPI) automated TEER measurement system (see key resources table). A WPI REMS-96C recording electrode was used to record TEER, which is compatible for use with a 96-well plate from Corning. The REMS-96C recording electrode was sterilized in 70% ethanol, followed by a rinse in PBS, and subsequently in media. The REMS-96C apical electrode was calibrated to measure TEER approximately 1 mm above the transwell membrane. Transwell-read time set to 5 s/well. Once set-up is complete, the plate is removed from incubator and TEER is measured directly afterward. To mitigate TEER artifacts due to temperature fluctuations, the same read sequence is repeated every subsequent read. TEER recorded by REMS AutoSampler were saved as.txt files; raw TEER values (in Ωs), are converted to normalized TEER values by Raw TEER in ohms (Ω) x surface area of transwell in cm2 = ohms. cm2 (SA = 0.143 cm2 for 96-well and 0.33 cm2 for 24-well).

##### Assessment of barrier permeability of EDMs using FITC-dextran

EDMs were grown for 48 h in 5% CM on 96-well transwells and TEER was monitored by a pair of REMS-96C recording electrodes using the WPI automated TEER Measurement System. After 48 h of growth, FITC-dextran (10 kD) was added to the apical side at a 1:50 dilution in 5% conditioned media. After 1 h of incubation with FITC-dextran, 50 μL of the basolateral supernatant was transferred to an opaque-black 96-well plate. Fluorescence was measured using excitation/emission 485 nm/535 nm with a Spark 20M Multimode Microplate Reader (see key resources table).

##### RNA isolation

Organoids and monolayers were lysed using 200 μl of RNA lysis buffer followed by RNA extraction per Zymo Research Quick-RNA MicroPrep Kit instructions (see key resources table). Tissue samples were lysed in TRI-Reagent and RNA was extracted using Zymo Research Direct-zol RNA Miniprep.

##### Quantitative (q)RT-PCR

Organoid and monolayer gene expression was measured by qRT-PCR using 2x SYBR Green qPCR Master Mix. cDNA was amplified with gene-specific primer/probe set and qScript cDNA SuperMix (5x). qRT-PCR was performed with the Applied Biosystems QuantStudio 5 Real-Time PCR System. Cycling parameters were as follows: 95°C for 20 s, followed by 40 cycles of 1 s at 95°C and 20 s at 60°C. Primers used in qRT-qPCR were previously validated in similar studies^95^^,^^96^^,^^97^ and primer sequences are stated in the Table S6^.^ All samples were assayed in triplicate and eukaryotic 18S ribosomal RNA was used as a reference.

##### DNA isolation

Organoid pellets were lysed using Genomic Lysis Buffer followed by DNA extraction per Zymo Research Quick-DNA Microprep Kit (see key resources table).

##### Genomic analysis of patient-derived organoids

A list of ∼154K SNPs associated with IBD was created by searching published papers,^98^^,^^99^^,^^100^ Clinvar and IBD genetics databases. A custom primer panel was developed by Tecan Genomics, Inc. (110053-192 Allegro Targeted Genotyping V2 50-100k; 10053-384 Allegro Targeted Genotyping V2 50-100k). DNA from CD and healthy-derived organoids were sequenced per Tecan’s instructions at the UCSD IGM Genomics Center. The sequencing data was processed by Interval Bio (San Diego, CA), a partner of Tecan Genomics, so that total SNPs and SNPs of special interests could be counted. More specifically, Interval Bio provided the bioinformatics service to list the single nucleotide polymorphism of genotype calls and variant calls with the rsIDs.

##### Treatment of EDMs with probiotic and postbiotic agents

The single cells from non-stricturing non-penetrating (NSNP) patient derived-organoids were seeded in 24-well transwells and allowed to differentiate for 2 days such that the TEER measurement (assessed manually, as described above) reached a stable plateau (indicative of maximal polarization of the monolayer)*. Escherichia coli* Nissle (EcN) was cultured using the same method as *AIEC-* LF82 and added to the apical side of the EDMs with a MOI of 100 and allowed to co-incubate for 8 h. The TEER was measured before and after the treatment at regular time intervals. To determine the impact of postbiotic Hylak Forte (HF), EDMs were preincubated for 12 h with HF and the TEER measurements were carried out for 24 h post-treatment at regular time intervals.

##### Assessment of DNA damage by flow cytometry

DNA double-strand breaks (DSBs) were measured by flow cytometry by detecting *γ*-H2AX in organoids after modifying the published methods.^101^ Briefly, single cell suspensions from healthy and CD colonic organoids were washed using FACS buffer (PBS, 5% FBS, 2 mM Sodium Azide) and fixed/stained using the Biolegend Cyto-Fast Fix Perm buffer set according to the manufacturer’s instructions. Cells were washed in Cyto-Fast Perm wash solution before incubation with the primary antibody anti-g*H2AX* (1:100) for 30 min at room temperature. Cells were washed with Cyto-Fast Perm wash solution, followed by incubation with secondary antibody Alexa Fluor 488 conjugated goat anti-mouse IgG and propidium iodide for 30 min in the dark at room temperature. Cells were washed with Cyto-Fast Perm Wash Solution, resuspended in FACS buffer and data was acquired on a Guava easyCyte flow cytometer (see key resources table). Data was analyzed using the FlowJo software (see key resources table).

##### Assessment of cellular senescence using SPIDER β-Galactosidase assay

Senescence associated β-Galactosidase (SA-βGal) was measured in healthy and CD colonic organoids following published protocols.^75^ Briefly, organoids were cultured for 7 days and incubated with the 100nM Bafilomycin A1 for 1 h to inhibit endogenous β-galactosidase activity. Organoids were washed using HBSS (without Ca2+/Mg2+) and incubated with 1 μM of SPIDER-βGal diluted in HBSS for 60 min at 37°C. Organoid were again washed with HBSS (without Ca2+/Mg2+) and disassociated to single cell suspensions with TrypLE and followed by filtering through 70 μm cell strainer and cells were immediately acquired on a Guava easyCyte flow cytometer (see key resources table). Data was analyzed using FlowJo software (see key resources table).

##### Multiplex immunoassays for quantification of cytokines

The cytokines were quantified from the supernatants collected from organoids and EDMs using customized Meso Scale Discovery (MSD)V-PLEX cytokine panels as per the manufacturer’s instructions. All data was obtained using a MESO QuickPlex SQ 120 instrument (see key resources table) and analyzed using MSD DISCOVERY WORKBENCH 4.0 software (see key resources table).

##### Bacterial clearance assay in CD colonic organoids and the impact of PPAR α/γ dual agonist

Adherent Invasive *Escherichia coli* strain LF82 (AIEC-*LF82*), isolated from the specimens of Crohn’s disease patient, was obtained from Arlette Darfeuille-Michaud.^77^ Bacterial clearance assay was performed following our published work.^22^^,^^51^ AIEC-*LF82* was grown in LB broth for 8 h under aerobic conditions and then under oxygen-limiting conditions overnight. Healthy and gray cluster EDMs were infected apically with AIEC-*LF82* with a multiplicity of infection (MOI) of 30 for 3 h in presence or absence of 1 μM PPAR α/γ dual agonist (PAR5359) with 16 h preincubation of the drug prior to infection. Gentamicin protection assaywas performed after 3 h with 200 μg/mL of gentamicin for 90 min, followed by serial dilution in 1x PBS and plating on LB agar plates. Colonies were counted the next day to measure colony forming unit per mL (cfu/mL).

#### Computational and bioinformatics approach

##### RNA sequencing and data processing

Raw FASTQ files were trimmed, filtered, and mapped to human genome for downstream quantification analyses. Low-quality sequences were trimmed or removed with Trimmomatic. STAR (version 2.6.0a) was used to align reads on the reference genome (human genome hsGRCh38_94). The resulting transcriptome-aligned sequences were used for expression quantification by using RSEM (version 1.3.3) with “–forward-prob 0” option. TPM scores for each sample were used through the RSEM tables (RSEM gene.results tables). We used log2(TPM) if TPM >1 else TPM - 1 values for each sample as the final gene expression value for downstream expression analyses.

##### RNASeq data analysis

RNASeq data was processed using traditional differential expression analysis tools (DESeq2), clustering techniques (PCA, Hierarchical Agglomerative Clustering), and visualized using heatmaps on R statistical software version 4.1.0 (2021-05-18). Principal component analysis (PCA) was performed in all samples based on bulk RNA seq data using *FactoMineR::PCA* function from R package:*FactoMineR*. Clusters of correlated variables were discovered using Hierarchical Clustering on Principal Components (*FactoMineR::HCPC*) analysis and highlighted using a factorial map (*FactoMineR* v2.5; *factoextra* v1.0.7; *ggplot2* v3.3.5). HCPC dendrograms were visually analyzed to find suitable number of clusters. Heatmaps with dendrograms were generated using python *clustermap* function from the *seaborn* package. The sample clusters description by genes (Figure S3) is established using *catdes* function from *FactoMineR* which performs a simple Chi-Square test with significance threshold = 0.05.

##### StepMiner analysis

StepMiner is an algorithm that identifies stepwise transitions using step function in a time-series data.^102^ StepMiner undergoes an adaptive regression scheme to verify the best possible up and down steps based on sum-of-square errors. The steps are placed between time points at the sharpest change between expression levels, which gives us the information about timing of the gene expression-switching event. In order to fit a step function, the algorithm evaluates all possible steps for each position and computes the average of the values on both sides of a step for the constant segments. An adaptive regression scheme is used that chooses the step positions that minimize the square error with the fitted data. Finally, a regression test statistic is computed as follows:

$$F\;stat=\frac{\sum_{i=1}^{n}{\left(\hat{X_{i}}\;-\;\bar{X}\right)}^{2}/\left(m-1\right)}{\sum_{i=1}^{n}{\left(X_{i}-\;\hat{X_{i}}\right)}^{2}/\left(n-m\right)}$$

Where $X_{i}$ for i=1 to n are the values, $\hat{X_{i}}$ for i=1 to n are fitted values. m is the degrees of freedom used for the adaptive regression analysis. $\bar{X}$ is the average of all the values: $\bar{X}=\frac{1}{n}*\;\sum_{j=1}^{n}X_{j}\text{.}$ For a step position at k, the fitted values $\hat{X_{l}}$ are computed by using $\frac{1}{k}*\;\sum_{j=1}^{n}X_{j}$ for i=1 to k and $\frac{1}{\left(n-k\right)}*\;\sum_{j=k+1}^{n}X_{j}$ for i=k+1 to n.

##### Composite gene signature analysis using Boolean network explorer (BoNE)

Boolean network explorer (BoNE) provides an integrated platform for the construction, visualization and querying of a gene expression signature underlying a disease or a biological process in three steps^20^: First, the expression levels of all genes in these datasets were converted to binary values (high or low) using the StepMiner algorithm. Second, Gene expression values were normalized according to a modified *Z* score approach centered around *StepMiner* threshold (formula = (expr - SThr)/3∗stddev). Third, the normalized expression values for every genes were added together to create the final score for the gene signature. The samples were ordered based on the final signature score. Classification of sample categories using this ordering is measured by ROC-AUC (Receiver Operating Characteristics Area Under The Curve) values. Welch’s Two Sample t-test (unpaired, unequal variance (equal_var = False), and unequal sample size) parameters were used to compare the differential signature score in different sample categories. Violin, Swarm and Bubble plots are created using python seaborn package version 0.10.1. Pathway analysis of gene lists were carried out via the Reactome database and algorithm.^90^

### Quantification and statistical analysis

All experiments were repeated at least three times, and results were presented either as one representative experiment (when images are displayed) or as average ±S.E.M (when displayed as graphs). Statistical significance between datasets with three or more experimental groups was determined either using one-way ANOVA including a Tukey’s test for multiple comparisons, or t-tests (Welch’s or Mann-Whitney), as indicated. For all tests, a P-value of 0.05 was used as the cutoff to determine significance and the real *p*-values are indicated in each figure. For all experiments, the statistical analyses were performed using GraphPad prism 6.1.

For computational analyses, the statistical tests were performed using R version 3.2.3 (2015-12-10). Standard t-tests were performed using python scipy.stats.ttest_ind package (version 0.19.0).

## Supplemental information

Document S1. Figures S1–S12

Table S1. Excel datasheet with the list of healthy and CD patients who were enrolled into this study and their demographic information, related to Figure 1

Table S2. Excel datasheet with the list of healthy and CD-PDOs that were analyzed in various functional assays, related to Figures 2–4

Table S3. Excel datasheet with the list of healthy and CD-PDOs that were analyzed by RNA-seq, related to Figure 2

Table S4. Excel datasheet with the list of differentially regulated genes in CD PDOs and their reactome pathway analyses, related to Figure 2

Table S5. Excel datasheet with gene list and rsIDs used for genotype analysis on CD PDOs, related to Figure 2

Table S6. Excel datasheet with qPCR primer sequences, related to Figure 4

Table S7. Excel datasheet with source data for cytokine analysis by MSD, related to Figure 5

Document S2. Article plus supplemental information

## References

[1] Baumgart D.C., Le Berre C. (2021). Newer Biologic and Small-Molecule Therapies for Inflammatory Bowel Disease. N. Engl. J. Med..

[2] Torres J., Mehandru S., Colombel J.F., Peyrin-Biroulet L. (2017). Crohn's disease. Lancet.

[3] Torres J., Colombel J.F. (2016). Genetics and phenotypes in inflammatory bowel disease. Lancet.

[4] Elmentaite R., Ross A.D.B., Roberts K., James K.R., Ortmann D., Gomes T., Nayak K., Tuck L., Pritchard S., Bayraktar O.A. (2020). Single-Cell Sequencing of Developing Human Gut Reveals Transcriptional Links to Childhood Crohn's Disease. Dev. Cell.

[5] Lee C., An M., Joung J.G., Park W.Y., Chang D.K., Kim Y.H., Hong S.N. (2022). TNFα Induces LGR5+ Stem Cell Dysfunction In Patients With Crohn's Disease. Cell. Mol. Gastroenterol. Hepatol..

[6] d'Aldebert E., Quaranta M., Sébert M., Bonnet D., Kirzin S., Portier G., Duffas J.P., Chabot S., Lluel P., Allart S. (2020). Characterization of Human Colon Organoids From Inflammatory Bowel Disease Patients. Front. Cell Dev. Biol..

[7] Arnauts K., Verstockt B., Ramalho A.S., Vermeire S., Verfaillie C., Ferrante M. (2020). Ex Vivo Mimicking of Inflammation in Organoids Derived From Patients With Ulcerative Colitis. Gastroenterology.

[8] Niklinska-Schirtz B.J., Venkateswaran S., Anbazhagan M., Kolachala V.L., Prince J., Dodd A., Chinnadurai R., Gibson G., Denson L.A., Cutler D.J. (2021). Ileal Derived Organoids From Crohn's Disease Patients Show Unique Transcriptomic and Secretomic Signatures. Cell. Mol. Gastroenterol. Hepatol..

[9] Østvik A.E., Svendsen T.D., Granlund A.V.B., Doseth B., Skovdahl H.K., Bakke I., Thorsvik S., Afroz W., Walaas G.A., Mollnes T.E. (2020). Intestinal Epithelial Cells Express Immunomodulatory ISG15 During Active Ulcerative Colitis and Crohn's Disease. J. Crohns Colitis.

[10] Kanke M., Kennedy Ng M.M., Connelly S., Singh M., Schaner M., Shanahan M.T., Wolber E.A., Beasley C., Lian G., Jain A. (2022). Single-cell analysis reveals unexpected cellular changes and transposon expression signatures in the colonic epithelium of treatment-naïve adult Crohn's disease patients. Cell. Mol. Gastroenterol. Hepatol..

[11] Chakravarti D., Lee R., Multani A.S., Santoni A., Keith Z., Hsu W.H., Chang K., Reyes L., Rashid A., Wu C.J. (2021). Telomere dysfunction instigates inflammation in inflammatory bowel disease. Proc. Natl. Acad. Sci. USA.

[12] Woznicki J.A., Saini N., Flood P., Rajaram S., Lee C.M., Stamou P., Skowyra A., Bustamante-Garrido M., Regazzoni K., Crawford N. (2021). TNF-α synergises with IFN-γ to induce caspase-8-JAK1/2-STAT1-dependent death of intestinal epithelial cells. Cell Death Dis..

[13] Lee C., Hong S.N., Kim E.R., Chang D.K., Kim Y.H. (2021). Epithelial Regeneration Ability of Crohn's Disease Assessed Using Patient-Derived Intestinal Organoids. Int. J. Mol. Sci..

[14] Xu P., Elizalde M., Masclee A., Pierik M., Jonkers D. (2021). Corticosteroid enhances epithelial barrier function in intestinal organoids derived from patients with Crohn's disease. J. Mol. Med..

[15] Xu P., Becker H., Elizalde M., Pierik M., Masclee A., Jonkers D. (2021). Interleukin-28A induces epithelial barrier dysfunction in CD patient-derived intestinal organoids. Am. J. Physiol. Gastrointest. Liver Physiol..

[16] Satsangi J., Silverberg M.S., Vermeire S., Colombel J.F. (2006). The Montreal classification of inflammatory bowel disease: controversies, consensus, and implications. Gut.

[17] Shehab M., Alrashed F., Heron V., Restellini S., Bessissow T. (2023). Comparative Efficacy of Biologic Therapies for Inducing Response and Remission in Fistulizing Crohn's Disease: Systematic Review and Network Meta-Analysis of Randomized Controlled Trials. Inflamm. Bowel Dis..

[18] Mills S., Stamos M.J. (2007). Colonic Crohn's disease. Clin. Colon Rectal Surg..

[19] Miyoshi H., Stappenbeck T.S. (2013). In vitro expansion and genetic modification of gastrointestinal stem cells in spheroid culture. Nat. Protoc..

[20] Sahoo D., Swanson L., Sayed I.M., Katkar G.D., Ibeawuchi S.R., Mittal Y., Pranadinata R.F., Tindle C., Fuller M., Stec D.L. (2021). Artificial intelligence guided discovery of a barrier-protective therapy in inflammatory bowel disease. Nat. Commun..

[21] Sayed I.M., Suarez K., Lim E., Singh S., Pereira M., Ibeawuchi S.R., Katkar G., Dunkel Y., Mittal Y., Chattopadhyay R. (2020). Host engulfment pathway controls inflammation in inflammatory bowel disease. FEBS J..

[22] Sayed I.M., Tindle C., Fonseca A.G., Ghosh P., Das S. (2021). Functional assays with human patient-derived enteroid monolayers to assess the human gut barrier. STAR Protoc..

[23] VanDussen K.L., Sonnek N.M., Stappenbeck T.S. (2019). L-WRN conditioned medium for gastrointestinal epithelial stem cell culture shows replicable batch-to-batch activity levels across multiple research teams. Stem Cell Res..

[24] Criss Z.K., Bhasin N., Di Rienzi S.C., Rajan A., Deans-Fielder K., Swaminathan G., Kamyabi N., Zeng X.L., Doddapaneni H., Menon V.K. (2021). Drivers of transcriptional variance in human intestinal epithelial organoids. Physiol. Genom..

[25] Sato T., Stange D.E., Ferrante M., Vries R.G.J., Van Es J.H., Van den Brink S., Van Houdt W.J., Pronk A., Van Gorp J., Siersema P.D., Clevers H. (2011). Long-term expansion of epithelial organoids from human colon, adenoma, adenocarcinoma, and Barrett's epithelium. Gastroenterology.

[26] Pizarro T.T., Stappenbeck T.S., Rieder F., Rosen M.J., Colombel J.F., Donowitz M., Towne J., Mazmanian S.K., Faith J.J., Hodin R.A. (2019). Challenges in IBD Research: Preclinical Human IBD Mechanisms. Inflamm. Bowel Dis..

[27] Korinek V., Barker N., Moerer P., van Donselaar E., Huls G., Peters P.J., Clevers H. (1998). Depletion of epithelial stem-cell compartments in the small intestine of mice lacking Tcf-4. Nat. Genet..

[28] Kuhnert F., Davis C.R., Wang H.T., Chu P., Lee M., Yuan J., Nusse R., Kuo C.J. (2004). Essential requirement for Wnt signaling in proliferation of adult small intestine and colon revealed by adenoviral expression of Dickkopf-1. Proc. Natl. Acad. Sci. USA.

[29] Pinto D., Gregorieff A., Begthel H., Clevers H. (2003). Canonical Wnt signals are essential for homeostasis of the intestinal epithelium. Genes Dev..

[30] Barker N., van Es J.H., Kuipers J., Kujala P., van den Born M., Cozijnsen M., Haegebarth A., Korving J., Begthel H., Peters P.J., Clevers H. (2007). Identification of stem cells in small intestine and colon by marker gene Lgr5. Nature.

[31] Sæterstad S., Østvik A.E., Røyset E.S., Bakke I., Sandvik A.K., Granlund A.V.B. (2022). Profound gene expression changes in the epithelial monolayer of active ulcerative colitis and Crohn's disease. PLoS One.

[32] Skovdahl H.K., Gopalakrishnan S., Svendsen T.D., Granlund A.V.B., Bakke I., Ginbot Z.G., Thorsvik S., Flatberg A., Sporsheim B., Ostrop J. (2021). Patient Derived Colonoids as Drug Testing Platforms-Critical Importance of Oxygen Concentration. Front. Pharmacol..

[33] Gopalakrishnan S., Hansen M.D., Skovdahl H.K., Roseth I.A., van Beelen Granlund A., Østvik A.E., Bakke I., Sandvik A.K., Bruland T. (2022). Tofacitinib Downregulates TNF and Poly(I:C)-Dependent MHC-II Expression in the Colonic Epithelium. Front. Immunol..

[34] Sarvestani S.K., Signs S., Hu B., Yeu Y., Feng H., Ni Y., Hill D.R., Fisher R.C., Ferrandon S., DeHaan R.K. (2021). Induced organoids derived from patients with ulcerative colitis recapitulate colitic reactivity. Nat. Commun..

[35] Di Marco Barros R., Roberts N.A., Dart R.J., Vantourout P., Jandke A., Nussbaumer O., Deban L., Cipolat S., Hart R., Iannitto M.L. (2016). Epithelia Use Butyrophilin-like Molecules to Shape Organ-Specific γδ T Cell Compartments. Cell.

[36] Nanki K., Fujii M., Shimokawa M., Matano M., Nishikori S., Date S., Takano A., Toshimitsu K., Ohta Y., Takahashi S. (2020). Somatic inflammatory gene mutations in human ulcerative colitis epithelium. Nature.

[37] Kakiuchi N., Yoshida K., Uchino M., Kihara T., Akaki K., Inoue Y., Kawada K., Nagayama S., Yokoyama A., Yamamoto S. (2020). Frequent mutations that converge on the NFKBIZ pathway in ulcerative colitis. Nature.

[38] Olafsson S., McIntyre R.E., Coorens T., Butler T., Jung H., Robinson P.S., Lee-Six H., Sanders M.A., Arestang K., Dawson C. (2020). Somatic Evolution in Non-neoplastic IBD-Affected Colon. Cell.

[39] Onichtchouk D., Chen Y.G., Dosch R., Gawantka V., Delius H., Massagué J., Niehrs C. (1999). Silencing of TGF-beta signalling by the pseudoreceptor BAMBI. Nature.

[40] Gusti V., Bennett K.M., Lo D.D. (2014). CD137 signaling enhances tight junction resistance in intestinal epithelial cells. Phys. Rep..

[41] Parikh K., Antanaviciute A., Fawkner-Corbett D., Jagielowicz M., Aulicino A., Lagerholm C., Davis S., Kinchen J., Chen H.H., Alham N.K. (2019). Colonic epithelial cell diversity in health and inflammatory bowel disease. Nature.

[42] Biton M., Haber A.L., Rogel N., Burgin G., Beyaz S., Schnell A., Ashenberg O., Su C.W., Smillie C., Shekhar K. (2018). T Helper Cell Cytokines Modulate Intestinal Stem Cell Renewal and Differentiation. Cell.

[43] Chakravarti D., Hu B., Mao X., Rashid A., Li J., Li J., Liao W.T., Whitley E.M., Dey P., Hou P. (2020). Telomere dysfunction activates YAP1 to drive tissue inflammation. Nat. Commun..

[44] Scaringi S., Di Martino C., Zambonin D., Fazi M., Canonico G., Leo F., Ficari F., Tonelli F. (2013). Colorectal cancer and Crohn's colitis: clinical implications from 313 surgical patients. World J. Surg..

[45] Biancone L., Armuzzi A., Scribano M.L., D'Inca R., Castiglione F., Papi C., Angelucci E., Daperno M., Mocciaro F., Riegler G. (2016). Inflammatory Bowel Disease Phenotype as Risk Factor for Cancer in a Prospective Multicentre Nested Case-Control IG-IBD Study. J. Crohns Colitis.

[46] Ikeuchi H., Nakano H., Uchino M., Nakamura M., Matsuoka H., Fukuda Y., Matsumoto T., Takesue Y., Tomita N. (2008). Intestinal cancer in Crohn's disease. Hepato-Gastroenterology.

[47] Sobala A., Herbst F., Novacek G., Vogelsang H. (2010). Colorectal carcinoma and preceding fistula in Crohn's disease. J. Crohns Colitis.

[48] Shaw K.A., Cutler D.J., Okou D., Dodd A., Aronow B.J., Haberman Y., Stevens C., Walters T.D., Griffiths A., Baldassano R.N. (2019). Genetic variants and pathways implicated in a pediatric inflammatory bowel disease cohort. Gene Immun..

[49] Meir M., Burkard N., Ungewiß H., Diefenbacher M., Flemming S., Kannapin F., Germer C.T., Schweinlin M., Metzger M., Waschke J., Schlegel N. (2019). Neurotrophic factor GDNF regulates intestinal barrier function in inflammatory bowel disease. J. Clin. Invest..

[50] Lacher M., Fitze G., Helmbrecht J., Schroepf S., Berger M., Lohse P., Koletzko S., Ballauff A., Grote V., Goedeke J. (2010). Hirschsprung-associated enterocolitis develops independently of NOD2 variants. J. Pediatr. Surg..

[51] Sharma A., Lee J., Fonseca A.G., Moshensky A., Kothari T., Sayed I.M., Ibeawuchi S.R., Pranadinata R.F., Ear J., Sahoo D. (2021). E-cigarettes compromise the gut barrier and trigger inflammation. iScience.

[52] Ghosh P., Swanson L., Sayed I.M., Mittal Y., Lim B.B., Ibeawuchi S.R., Foretz M., Viollet B., Sahoo D., Das S. (2020). The stress polarity signaling (SPS) pathway serves as a marker and a target in the leaky gut barrier: implications in aging and cancer. Life Sci. Alliance.

[53] Schoultz I., Keita Å.V. (2020). The Intestinal Barrier and Current Techniques for the Assessment of Gut Permeability. Cells.

[54] Srinivasan B., Kolli A.R., Esch M.B., Abaci H.E., Shuler M.L., Hickman J.J. (2015). TEER measurement techniques for in vitro barrier model systems. J. Lab. Autom..

[55] Grondin J.A., Kwon Y.H., Far P.M., Haq S., Khan W.I. (2020). Mucins in Intestinal Mucosal Defense and Inflammation: Learning From Clinical and Experimental Studies. Front. Immunol..

[56] Dincer Y., Erzin Y., Himmetoglu S., Gunes K.N., Bal K., Akcay T. (2007). Oxidative DNA damage and antioxidant activity in patients with inflammatory bowel disease. Dig. Dis. Sci..

[57] Jang W.H., Park A., Wang T., Kim C.J., Chang H., Yang B.G., Kim M.J., Myung S.J., Im S.H., Jang M.H. (2018). Two-photon microscopy of Paneth cells in the small intestine of live mice. Sci. Rep..

[58] Farin H.F., Karthaus W.R., Kujala P., Rakhshandehroo M., Schwank G., Vries R.G.J., Kalkhoven E., Nieuwenhuis E.E.S., Clevers H. (2014). Paneth cell extrusion and release of antimicrobial products is directly controlled by immune cell-derived IFN-γ. J. Exp. Med..

[59] Pullan R.D., Thomas G.A., Rhodes M., Newcombe R.G., Williams G.T., Allen A., Rhodes J. (1994). Thickness of adherent mucus gel on colonic mucosa in humans and its relevance to colitis. Gut.

[60] Rhodes J.M. (1997). Mucins and inflammatory bowel disease. QJM.

[61] Sasaki T., Hiwatashi N., Yamazaki H., Noguchi M., Toyota T. (1992). The role of interferon gamma in the pathogenesis of Crohn's disease. Gastroenterol. Jpn..

[62] Yu S., Balasubramanian I., Laubitz D., Tong K., Bandyopadhyay S., Lin X., Flores J., Singh R., Liu Y., Macazana C. (2020). Paneth Cell-Derived Lysozyme Defines the Composition of Mucolytic Microbiota and the Inflammatory Tone of the Intestine. Immunity.

[63] Singh R., Balasubramanian I., Zhang L., Gao N. (2020). Metaplastic Paneth Cells in Extra-Intestinal Mucosal Niche Indicate a Link to Microbiome and Inflammation. Front. Physiol..

[64] Tanaka M., Saito H., Kusumi T., Fukuda S., Shimoyama T., Sasaki Y., Suto K., Munakata A., Kudo H. (2001). Spatial distribution and histogenesis of colorectal Paneth cell metaplasia in idiopathic inflammatory bowel disease. J. Gastroenterol. Hepatol..

[65] Fahlgren A., Hammarström S., Danielsson A., Hammarström M.L. (2003). Increased expression of antimicrobial peptides and lysozyme in colonic epithelial cells of patients with ulcerative colitis. Clin. Exp. Immunol..

[66] Moran G., McLaughlin J. (2011). Enteroendocrine cells and appetite dysregulation in Crohn's disease. Gut.

[67] Hunziker W., Spiess M., Semenza G., Lodish H.F. (1986). The sucrase-isomaltase complex: primary structure, membrane-orientation, and evolution of a stalked, intrinsic brush border protein. Cell.

[68] Beaulieu J.F., Weiser M.M., Herrera L., Quaroni A. (1990). Detection and characterization of sucrase-isomaltase in adult human colon and in colonic polyps. Gastroenterology.

[69] Blander J.M. (2016). Death in the intestinal epithelium-basic biology and implications for inflammatory bowel disease. FEBS J..

[70] Holt P.R., Moss S.F., Kapetanakis A.M., Petrotos A., Wang S. (1997). Is Ki-67 a better proliferative marker in the colon than proliferating cell nuclear antigen?. Cancer Epidemiol. Biomarkers Prev..

[71] Noffsinger A., Unger B., Fenoglio-Preiser C.M. (1998). Increased cell proliferation characterizes Crohn's disease. Mod. Pathol..

[72] Weber A., Marques-Maggio E. (2013). Apoptotic colonopathy under immunosuppression: mycophenolate-related effects and beyond. Pathobiology.

[73] Talmon G., Manasek T., Miller R., Muirhead D., Lazenby A. (2017). The Apoptotic Crypt Abscess: An Underappreciated Histologic Finding in Gastrointestinal Pathology. Am. J. Clin. Pathol..

[74] Schulzke J.D., Bojarski C., Zeissig S., Heller F., Gitter A.H., Fromm M. (2006). Disrupted barrier function through epithelial cell apoptosis. Ann. N. Y. Acad. Sci..

[75] Tummon I.S., Maclin V.M., Radwanska E., Binor Z., Dmowski W.P. (1988). Occult ovulatory dysfunction in women with minimal endometriosis or unexplained infertility. Fertil. Steril..

[76] Xu M., Tchkonia T., Ding H., Ogrodnik M., Lubbers E.R., Pirtskhalava T., White T.A., Johnson K.O., Stout M.B., Mezera V. (2015). JAK inhibition alleviates the cellular senescence-associated secretory phenotype and frailty in old age. Proc. Natl. Acad. Sci. USA.

[77] Darfeuille-Michaud A., Boudeau J., Bulois P., Neut C., Glasser A.L., Barnich N., Bringer M.A., Swidsinski A., Beaugerie L., Colombel J.F. (2004). High prevalence of adherent-invasive Escherichia coli associated with ileal mucosa in Crohn's disease. Gastroenterology.

[78] Kim M.K., Chae Y.N., Son M.H., Kim S.H., Kim J.K., Moon H.S., Park C.S., Bae M.H., Kim E., Han T. (2008). PAR-5359, a well-balanced PPARalpha/gamma dual agonist, exhibits equivalent antidiabetic and hypolipidemic activities in vitro and in vivo. Eur. J. Pharmacol..

[79] Katkar G.D., Sayed I.M., Anandachar M.S., Castillo V., Vidales E., Toobian D., Usmani F., Sawires J.R., Leriche G., Yang J. (2021). Artificial Intelligence-rationalized balanced PPARα/γ dual agonism resets the dysregulated macrophage processes in inflammatory bowel disease. bioRxiv.

[80] Kruis W., Fric P., Pokrotnieks J., Lukás M., Fixa B., Kascák M., Kamm M.A., Weismueller J., Beglinger C., Stolte M. (2004). Maintaining remission of ulcerative colitis with the probiotic Escherichia coli Nissle 1917 is as effective as with standard mesalazine. Gut.

[81] Schultz M. (2008). Clinical use of E. coli Nissle 1917 in inflammatory bowel disease. Inflamm. Bowel Dis..

[82] Chakravarti D., DePinho R.A. (2021). Telomere Dysfunction as an Initiator of Inflammation: Clues to an Age-Old Mystery. J. Inflamm. Bowel Dis. Disord..

[83] Rogler G. (2020). Efficacy of JAK inhibitors in Crohn's Disease. J. Crohns Colitis.

[84] Finkbeiner S.R., Spence J.R. (2013). A gutsy task: generating intestinal tissue from human pluripotent stem cells. Dig. Dis. Sci..

[85] Arijs I., De Hertogh G., Lemaire K., Quintens R., Van Lommel L., Van Steen K., Leemans P., Cleynen I., Van Assche G., Vermeire S. (2009). Mucosal gene expression of antimicrobial peptides in inflammatory bowel disease before and after first infliximab treatment. PLoS One.

[86] Corraliza A.M., Ricart E., López-García A., Carme Masamunt M., Veny M., Esteller M., Mayorgas A., Le Bourhis L., Allez M., Planell N. (2019). Differences in Peripheral and Tissue Immune Cell Populations Following Haematopoietic Stem Cell Transplantation in Crohn's Disease Patients. J. Crohns Colitis.

[87] Verstockt B., Verstockt S., Dehairs J., Ballet V., Blevi H., Wollants W.J., Breynaert C., Van Assche G., Vermeire S., Ferrante M. (2019). Low TREM1 expression in whole blood predicts anti-TNF response in inflammatory bowel disease. EBioMedicine.

[88] Gào X., Holleczek B., Cuk K., Zhang Y., Anusruti A., Xuan Y., Xu Y., Brenner H., Schottker B. (2019). Investigation on potential associations of oxidatively generated DNA/RNA damage with lung, colorectal, breast, prostate and total cancer incidence. Sci. Rep..

[89] Rodrigues D.G.B., de Moura Coelho D., Sitta Â., Jacques C.E.D., Hauschild T., Manfredini V., Bakkali A., Struys E.A., Jakobs C., Wajner M., Vargas C.R. (2017). Experimental evidence of oxidative stress in patients with l-2-hydroxyglutaric aciduria and that l-carnitine attenuates in vitro DNA damage caused by d-2-hydroxyglutaric and l-2-hydroxyglutaric acids. Toxicol. Vitro.

[90] Fabregat A., Jupe S., Matthews L., Sidiropoulos K., Gillespie M., Garapati P., Haw R., Jassal B., Korninger F., May B. (2018). The Reactome Pathway Knowledgebase. Nucleic Acids Res..

[91] Sayed I.M., Sahan A.Z., Venkova T., Chakraborty A., Mukhopadhyay D., Bimczok D., Beswick E.J., Reyes V.E., Pinchuk I., Sahoo D. (2020). Helicobacter pylori infection downregulates the DNA glycosylase NEIL2, resulting in increased genome damage and inflammation in gastric epithelial cells. J. Biol. Chem..

[92] Sayed I.M., Chakraborty A., Abd El Hafeez A.A.,A.,S., Sahan A.Z., WJM H., Sahoo D., Ghosh P., Hazra T.K., Das S. (2020). The DNA Glycosylase NEIL2 Suppresses Fusobacterium-Infection-Induced Inflammation and DNA Damage in Colonic Epithelial Cells. Cells.

[93] Tindle C., Fuller M., Fonseca A., Taheri S., Ibeawuchi S.R., Beutler N., Katkar G.D., Claire A., Castillo V., Hernandez M. (2021). Adult stem cell-derived complete lung organoid models emulate lung disease in COVID-19. Elife.

[94] Lopez-Sanchez I., Dunkel Y., Roh Y.S., Mittal Y., De Minicis S., Muranyi A., Singh S., Shanmugam K., Aroonsakool N., Murray F. (2014). GIV/Girdin is a central hub for profibrogenic signalling networks during liver fibrosis. Nat. Commun..

[95] Molina-Castro S.E., Tiffon C., Giraud J., Boeuf H., Sifre E., Giese A., Belleannée G., Lehours P., Bessède E., Mégraud F. (2020). The Hippo Kinase LATS2 Controls Helicobacter pylori-Induced Epithelial-Mesenchymal Transition and Intestinal Metaplasia in Gastric Mucosa. Cell. Mol. Gastroenterol. Hepatol..

[96] Prakash R., Bharathi Raja S., Devaraj H., Devaraj S.N. (2011). Up-regulation of MUC2 and IL-1β expression in human colonic epithelial cells by Shigella and its interaction with mucins. PLoS One.

[97] Date K., Yamazaki T., Toyoda Y., Hoshi K., Ogawa H. (2020). α-Amylase expressed in human small intestinal epithelial cells is essential for cell proliferation and differentiation. J. Cell. Biochem..

[98] Jostins L., Ripke S., Weersma R.K., Duerr R.H., McGovern D.P., Hui K.Y., Lee J.C., Schumm L.P., Sharma Y., Anderson C.A. (2012). Host-microbe interactions have shaped the genetic architecture of inflammatory bowel disease. Nature.

[99] Liu J.Z., van Sommeren S., Huang H., Ng S.C., Alberts R., Takahashi A., Ripke S., Lee J.C., Jostins L., Shah T. (2015). Association analyses identify 38 susceptibility loci for inflammatory bowel disease and highlight shared genetic risk across populations. Nat. Genet..

[100] Peloquin J.M., Goel G., Kong L., Huang H., Haritunians T., Sartor R.B., Daly M.J., Newberry R.D., McGovern D.P., Yajnik V. (2016). Characterization of candidate genes in inflammatory bowel disease-associated risk loci. JCI Insight.

[101] Firsanov D., Solovjeva L., Lublinskaya O., Zenin V., Kudryavtsev I., Serebryakova M., Svetlova M. (2017). Rapid Detection of gamma-H2AX by Flow Cytometry in Cultured Mammalian Cells. Methods Mol. Biol..

[102] Sahoo D., Dill D.L., Tibshirani R., Plevritis S.K. (2007). Extracting binary signals from microarray time-course data. Nucleic Acids Res..

